# Electron acceptor-dependent duality of nitrous oxide metabolism in *Thiobacillus* during sulfur autotrophic denitrification

**DOI:** 10.3389/fmicb.2026.1903473

**Published:** 2026-09-04

**Authors:** Muchun Zhou, Shohei Yasuda, Hiroto Miura, Tianxiang Xu, Megumi Kuroiwa, Xiaoguang Xu, Akihiko Terada

**Affiliations:** 1 Department of Applied Physics and Chemical Engineering, Tokyo University of Agriculture and Technology, Tokyo, Japan; 2 Civil Engineering, School of Engineering, College of Science and Engineering, University of Galway, Galway, Ireland; 3 School of Environment, Nanjing Normal University, Nanjing, China

**Keywords:** clade I/II nosZ gene, electron acceptor, nitrous oxide, sulfur autotrophic denitrification, *Thiobacillus*

## Abstract

Sulfur-driven autotrophic denitrification (SADN) is a promising biotechnology for nitrogen removal from low-carbon wastewater; however, nitrous oxide (N_2_O) emissions remain a significant environmental concern. This study systematically investigated the effects of different nitrogen oxide electron acceptors on denitrification performance, microbial community succession, distribution of the N_2_O reductase gene *nosZ* clade, and the ecological functions of *Thiobacillus* using sequential enrichment cultivation. Among the tested conditions, the nitrate (${\text{NO}}_{3}^{-}$)-fed system achieved the highest denitrification and sulfur oxidation rates, with the lowest net N_2_O accumulation, whereas the nitrite (${\text{NO}}_{2}^{-}$)-fed condition led to severe N_2_O accumulation due to an imbalance between N_2_O production and reduction. High-throughput sequencing and quantitative PCR analyses revealed that *Thiobacillus* became the primary sulfur-oxidizing denitrifier in the presence of ${\text{NO}}_{3}^{-}$, ${\text{NO}}_{2}^{-}$, and nitric oxide, accompanied by substantial enrichment of *nirS* and clade I *nosZ* genes. In contrast, N_2_O-fed conditions promoted a more functionally diverse community enriched with clade II *nosZ* bacteria, including *Azonexus* and *Dechloromonas*. Metagenomic analyses recovered three distinct *Thiobacillus* metagenome-assembled genomes (MAGs 10, 11, and 25), each with distinct denitrification and sulfur oxidation capacities. MAG 10 contains genes for complete sulfur oxidation and denitrification, including clade I *nosZ* and *nirS* genes. Conversely, the *nosZ* gene was not detected in MAG 11, whereas the *norB/norC* genes were present, indicating their potential role as an N_2_O producer. MAG 25 exhibits N_2_O-responsive functional enrichment upon N_2_O feeding, reflecting a specialized ecological strategy centered on sulfur oxidation coupled with N_2_O reduction. Overall, these findings show that electron acceptors play a key role in shaping microbial succession, *nosZ* clade distribution, and the dual roles of *Thiobacillus* in N_2_O cycling depending on conditions. This study provides new insights into microbial ecology and offers potential strategies for mitigating N_2_O emissions in SADN processes.

## 1 Introduction

Nitrous oxide (N_2_O) is the third most abundant greenhouse gas (GHG) in the atmosphere and the most significant ozone-depleting substance currently emitted. It has an atmospheric lifetime of 116 years, which is 10 times longer than CH_4_, and a global warming potential 265 times that of CO_2_ (IPCC, 2022). Over the past four decades, N_2_O emissions from municipal and industrial wastewater have increased by 30% (Tian et al., 2020), raising concerns about their notable contribution to the greenhouse gas footprint of wastewater treatment plants. N_2_O accumulates during biological nitrogen removal from wastewater as both a byproduct and intermediate of nitrification and denitrification, respectively (Conthe et al., 2019). In particular, the demand for denitrification to reduce N_2_O emissions has risen due to stricter effluent nitrogen standards. Understanding the microbial functions of N_2_O production and consumption is crucial because these factors influence secondary pollution and N_2_O emissions (Liu et al., 2016; Chen et al., 2018; An et al., 2024). Therefore, achieving low levels of organic carbon and nitrogen in effluent while minimizing N_2_O emissions presents a dual challenge for wastewater treatment within the context of global climate change and water environment preservation.

To alleviate the contradiction between improving water quality and reducing GHG emissions, sulfur autotrophic denitrification (SADN) has emerged as a promising denitrification strategy in recent years (Sahinkaya and Dursun, 2015; Wang et al., 2023). SADN couples sulfur oxidation with nitrogen oxide reduction, utilizing reduced sulfur compounds, e.g., elemental sulfur, thiosulfate, and iron sulfide minerals, as electron donors and nitrogen oxides, e.g., nitrate (${\text{NO}}_{3}^{-}$) and nitrite (${\text{NO}}_{2}^{-}$), as electron acceptors, thereby effectively removing ${\text{NO}}_{3}^{-}$ from wastewater devoid of organic carbon (Bai et al., 2023). The SADN process does not require external organic carbon supplementation; thus, it can minimize excess sludge production, making it advantageous for treating wastewater containing nitrogen and sulfur devoid of organic carbon (Sahinkaya and Kilic, 2014). In addition, SADN significantly reduces N_2_O emissions, with an N_2_O conversion rate of 0.3%−0.6% for ${\text{NO}}_{3}^{-}$ removed in SADN (Zhang et al., 2015). Reportedly, gaseous N_2_O emission via SADN was 6–8 times lower than that via heterotrophic denitrification (Yang et al., 2016a; Wang et al., 2017). However, N_2_O emissions from SADN systems have received comparatively limited attention, and the microbial basis for N_2_O accumulation under different operational conditions remains poorly resolved.

Members of the genus *Thiobacillus* are widely recognized as key functional and dominant microorganisms among sulfur-oxidizing denitrifiers in SADN systems. These chemolithoautotrophic sulfur oxidizers can couple the oxidation of reduced sulfur compounds with the reduction of ${\text{NO}}_{3}^{-}$ or ${\text{NO}}_{2}^{-}$ (Beller et al., 2006a). Due to their robust sulfur-oxidation capabilities and adaptability to sulfur-rich environments, *Thiobacillus* species often predominate in engineered sulfur-based denitrification systems. Previous genomic and physiological studies have shown that certain *Thiobacillus* species possess complete denitrification pathways, including *nar, nir, nor*, and *nos* gene clusters (Beller et al., 2006b; Shao et al., 2010), allowing the sequential reduction of ${\text{NO}}_{3}^{-}$ to dinitrogen gas (Kelly and Wood, 2000a). As a result, *Thiobacillus* is frequently regarded as an important microbial sink for N_2_O in sulfur-driven denitrification.

However, increasing evidence suggests that the ecological role of *Thiobacillus* in N_2_O metabolism is considerably more complex than previously recognized. Previous studies have shown that the genus *Thiobacillus* can either produce or consume N_2_O depending on environmental conditions (Chen et al., 2020). Whether it functions as an N_2_O source or a sink depends on the sulfur supply and redox conditions. However, the precise mechanistic basis for this functional flexibility in N_2_O metabolism at the species level remains poorly understood. In particular, it is unclear whether the same *Thiobacillus* population alters N_2_O production and reduction activities in response to changes in gene expression, or whether different genetic distinct *Thiobacillus* lineages prevail under varying conditions. Investigating these possibilities is crucial for accurately predicting N_2_O emissions in SADN systems because each mechanism requires a distinct microbial control strategy. Moreover, whether N_2_O exerts selective pressures that shape the community composition of N_2_O producers and reducers in SADN systems remains unknown.

Recent advances in metagenomics and genome-resolved microbial ecology have enabled deeper insights into the functional diversity of denitrifying microorganisms beyond genus-level classification. Notably, the nitrous oxide reductase gene *nosZ* can be divided into three phylogenetically distinct clades: clade I *nosZ*, clade II *nosZ*, and clade III *nosZ* (Sanford et al., 2012; Hallin et al., 2018; He et al., 2025). Canonical sulfur-oxidizing denitrifiers such as *Thiobacillus* typically harbor clade I *nosZ* and denitrification genes for complete denitrification, whereas clade II *nosZ* is frequently associated with noncanonical or non-denitrifying N_2_O reducers that exhibit higher substrate affinity for N_2_O. Increasing evidence indicates that interactions between clade I and clade II *nosZ*-containing populations are essential for controlling N_2_O fluxes in engineered ecosystems (Conthe et al., 2018; Qi et al., 2022). However, there is limited knowledge regarding how different nitrogen oxide acceptors shape the enrichment of these *nosZ* clades and their ecological interactions in sulfur-driven autotrophic denitrification systems.

Therefore, the objectives of this study were to investigate how different nitrogen oxide electron acceptors influence sulfur-driven autotrophic denitrification performance, microbial community succession, *nosZ* clade distribution, and the functional differentiation of *Thiobacillus* populations during sequential enrichment cultivation. High-throughput 16S rRNA gene sequencing, quantitative PCR, and metagenomic analyses were integrated to elucidate the ecological and metabolic mechanisms underlying N_2_O production and consumption in SADN systems. Particular emphasis lay in the condition-dependent dual roles of *Thiobacillus* in N_2_O cycling and the complementary interactions between clade I and clade II *nosZ*-containing microorganisms. This study provides new insights into the microbial ecology of sulfur-driven autotrophic denitrification and offers potential strategies for mitigating N_2_O emissions in engineered sulfur-based nitrogen removal systems.

## 2 Materials and methods

### 2.1 Cultivation setup and operating conditions

Batch tests with different nitrogen oxides were performed to enrich sulfur-oxidizing denitrifiers in serum vials (100 mL) containing the headspace (50 mL), liquid medium (50 mL), and biomass (Supplementary Figure S1A). The bottles were statically incubated at 30 °C and kept in the dark to prevent photooxidation of iron and sulfide. Controls without biomass or electron acceptors were executed in triplicate to assess the impact of abiotic reactions on substrate concentrations and to evaluate the contribution of organic carbon present in biomass to endogenous denitrification. All enrichments were performed in triplicate using a gas-displacement device to fill the headspace with 99.99% helium (He). In the batch cultivation experiment, the initial set of bottles was inoculated with activated sludge from a municipal wastewater treatment plant. Subsequently, for the second set of subcultures, 20% (v/v) of the previous cultures was transferred to newly prepared vials whenever nitrogen compounds were completely depleted or when sulfide and sulfate concentrations reached the stationary phase, following the strategy described elsewhere (Carboni et al., 2021). All incubations were performed in triplicate. The third subculture was also performed following the same transfer protocol. However, due to substantial decreases in biomass concentrations, denitrification and sulfur oxidation activities deteriorated markedly across all four electron-acceptor conditions during this cycle. The third subculture was therefore excluded from all subsequent analyses of microbial community compositions, gene abundance and functions, and all results reported in this study correspond exclusively to the first and second subculture stages.

### 2.2 Electron donors and acceptors

The synthetic medium contained electron donors, electron acceptors, and a basic medium. The sole electron donor, reduced sulfur (S^2−^), was supplied by Na_2_S·9H_2_O, whereas the electron acceptors were ${\text{NO}}_{3}^{-}$, ${\text{NO}}_{2}^{-}$, nitric oxide (NO), and N_2_O. The experimental conditions, including the types and concentrations of electron donors and acceptors used, are summarized in Table 1. The details of medium compositions are given in Supplementary Table S1. For gaseous electron acceptors (NO and N_2_O), a gas-displacement device was used to replace the headspace air with helium. This procedure was followed by injecting the corresponding volume of nitrogen-containing compounds and retaining headspace pressure (~ 50 kPa) in each bottle.

**Table 1:** Feeding schemes of nitrogen oxides and sulfide.

Cultivation stage	Sample no.	Nitrogen oxides	Concentration (mg-N/L)	Sulfide (mg-S/L)
First subculture (S1 for 18 days)	S11	${\text{NO}}_{3}^{-}$	70	Initial concentration of 105 mg-S/L in each vial
	S12	${\text{NO}}_{2}^{-}$	117	
	S13	NO (gas)	175	
	S14	N_2_O (gas)	175	
Second subculture (S2 for 20 days)	S21	${\text{NO}}_{3}^{-}$	70	
	S22	${\text{NO}}_{2}^{-}$	117	
	S23	NO (gas)	175	
	S24	N_2_O (gas)	175	

### 2.3 Chemical analysis

The supernatant of each sample was filtered using a 0.45 μm membrane filter (A045A025A, Advantec, Tokyo, Japan) prior to water quality analyses. ${\text{NO}}_{3}^{-}$, ${\text{NO}}_{2}^{-}$, ${\text{SO}}_{4}^{2-}$, and ${\text{NH}}_{4}^{+}$ concentrations were measured using an ion chromatography system (ICS1000 and ICS90, ThermoFisher, Boston, MA). Gaseous N_2_O concentration in the headspace was quantified using a quadruple gas chromatography-mass spectrometer (GCMS-QP2010 Ultra, Shimadzu, Kyoto, Japan). Total dissolved S^2−^ concentrations were measured using the methylene blue method immediately after sampling (APHA, 2005). Dissolved oxygen (DO) concentration was monitored by a DO spot sensor (Optical Oxygen Monitor, FireSting O_2_-C, BAS), as shown in Supplementary Figure S1B. Mixed liquor suspended solids (MLSS) and mixed liquor volatile suspended solids (MLVSS) concentrations were measured following standard methods (APHA, 2005). pH values were measured using a portable pH meter (D-53, HORIBA, Kyoto, Japan) equipped with a pH sensor (9625-10D, HORIBA, Kyoto, Japan). The pH was adjusted to ~7.5 before subculturing.

Due to the high chemical reactivity and instability of NO in aqueous environments, including its rapid oxidation to ${\text{NO}}_{2}^{-}$ in the presence of trace oxygen (Schreiber et al., 2012; Ford and Miranda, 2020), direct measurement of NO was not performed in this study. Instead, ${\text{NO}}_{2}^{-}$ concentrations were monitored as an indirect indicator of NO transformation during the incubation. Accordingly, the NO treatment described in this study refers to the nominal NO dosage rather than the directly measured NO concentrations.

### 2.4 Activity analysis

Based on the methods described in the Supplementary material, we calculated biomass-specific denitrification and sulfur oxidation rates, as well as N_2_O accumulation and consumption rates for the first and second subcultures under different electron acceptor conditions. Given the rapid conversion of NO to ${\text{NO}}_{2}^{-}$ (Supplementary Figure S2), the initial NO concentration only had an effect at the start.

### 2.5 DNA extraction

Enriched biomass samples were collected for microbial community analyses at the end of each cultivation stage. DNA was extracted from the collected biomass using a FastDNA Spin Kit (MP Biochemicals, CA, United States). The concentration and purity of the extracted DNA were measured using a NanoDrop 2000c (Thermo Fisher Scientific, Waltham, MA), and the DNA was stored at −30 °C prior to further analysis.

### 2.6 16S rRNA gene amplicon sequencing analysis

Amplicon sequencing of the 16S rRNA gene was performed using the Illumina MiSeq sequencing platform (MiSeq Reagent Kit v3, Illumina, CA). The amplicons targeting the V4 hypervariable regions of the bacterial 16S rRNA gene were generated using a 2-step tailed PCR method (Supplementary Table S2). Library quality and concentration were assessed before sequencing. Sequencing was conducted in paired-end mode (2 × 300 bp) according to the manufacturer's protocol. Detailed protocols for DNA quantification, library preparation, quality assessment, and bioinformatics processing are provided in Supplementary material.

### 2.7 Quantitative PCR (qPCR) analysis

Functional genes encoding nitrite reductase (*nirS* and *nirK*) and N_2_O reductase (clade I *nosZ* and clade II *nosZ*) were quantified using a CFX96 Real-Time PCR Detection System (BioRad Laboratories, CA). Detailed information regarding the primer sets and PCR conditions is shown in Supplementary Table S3. Recently, the clade III *nosZ* was discovered (He et al., 2025). According to 16S rRNA gene amplicon sequencing, the genera affiliated with clade III *nosZ* were of marginal abundances. Hence, no quantification of the clade III *nosZ* was performed.

### 2.8 Metagenome data processing

DNA was extracted from four biomass samples (S21-S24) from the second cultivation and purified as previously reported (Zhou et al., 2025). Metagenomic analysis was conducted using the MetaWRAP v1.3.2 pipeline. Both co-assembly and single-assembly approaches were performed using MEGAHIT v1.1.3 to reconstruct microbial genomes from metagenomic reads. The overall workflow of the metagenomic analysis is illustrated (Supplementary Figure S3). Library preparation and sequencing were performed at the Bioengineering Lab. Co., Ltd. (Kanagawa, Japan). Sequencing was performed on a DNBSEQ-T7 (MGI Tech, Tokyo, Japan) using a 150-bp paired-end sequencing protocol, and 12 Gb of data was obtained per sample. Detailed information is shown in the Supplementary material.

### 2.9 Nucleotide accession number

The amplicon sequencing data based on the 16S rRNA have been deposited into the DDBJ Sequence Read Archive with the following accession numbers: DRA028838-DRA028846, SSUB042900, BioProject: PRJDB35817. The sequencing data generated by metagenomic analysis has been deposited into the DDBJ Sequence Read Archive with the following accession numbers: DRA028894-DRA028897, SSUB046959, BioProject: PRJDB35817.

### 2.10 Data analysis

All statistical analyses were performed using IBM SPSS Statistics (IBM Corp., Armonk, NY, United States). All figures, including the correlation plot, heatmap plot, and principal component analysis (PCA) plot, were generated using OriginPro (OriginLab Corporation, Northampton, MA, United States). Microbial community structure was analyzed using PCA to evaluate the dissimilarity among microbial communities across different electron acceptor conditions and subculture stages. The relationship between *Thiobacillus* relative abundance and denitrifying gene copy numbers was investigated using Spearman rank correlation analysis, with statistically significant correlations defined at *p* ≤ 0.05.

## 3 Results

### 3.1 Denitrification and sulfur oxidation rates of subcultures

**Figure 1:**
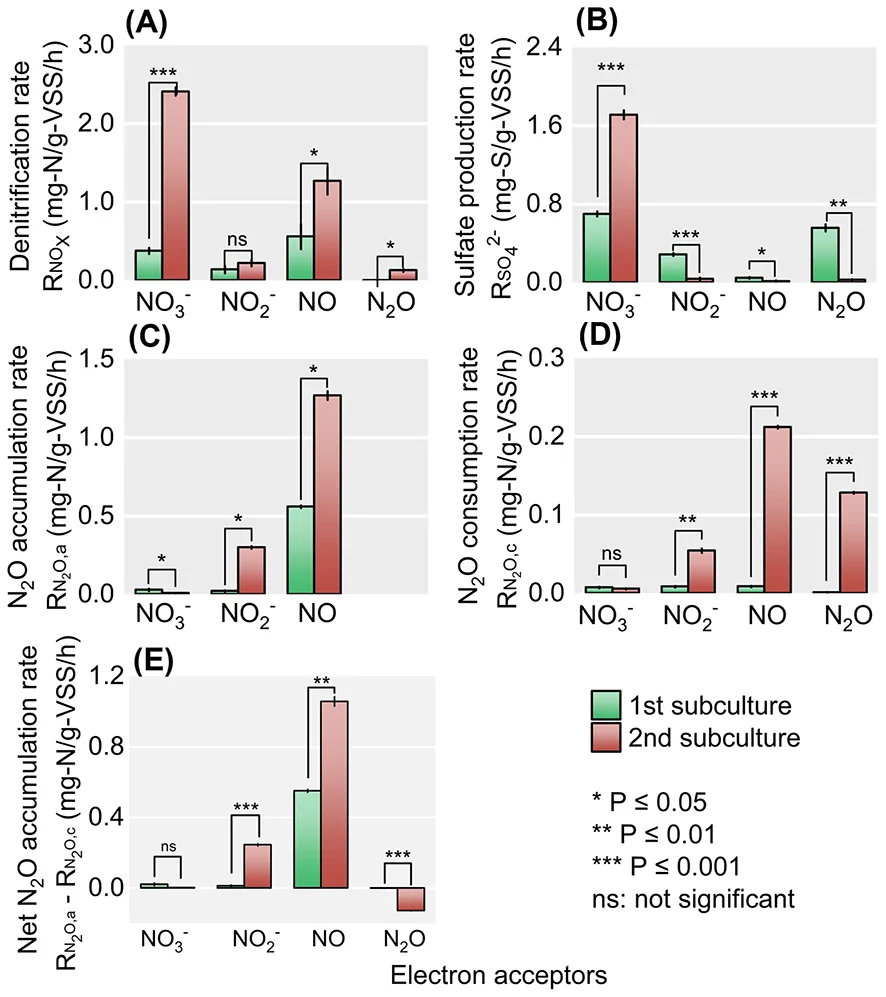
**(A)** The biomass-specific nitrogen oxide reduction rates (RNO_X_). **(B)** Sulfate production rates (${\text{RSO}}_{4}^{2-}$). **(C)** N_2_O accumulation rates (RN_2_O, a). **(D)** N_2_O consumption rates (RN_2_O, c). **(E)** Net N_2_O accumulation rates (RN_2_O, a – RN_2_O, c) in the first and second subcultures. Under N_2_O conditions, the denitrification rate equals the N_2_O consumption rate, and no N_2_O accumulation occurred during the experiment.

As shown in Figure 1A, subculturing markedly increased the ${\text{NO}}_{3}^{-}$ consumption rate from 0.38 mg-N/g-VSS/h after the first cultivation to 2.41 mg-N/g-VSS/h after the second cultivation. Similarly, the consumption rates of ${\text{NO}}_{2}^{-}$ and NO increased from 0.14 to 0.22 mg-N/g-VSS/h and from 0.56 to 1.27 mg-N/g-VSS/h, respectively. Whereas N_2_O, as the sole electron acceptor, the N_2_O consumption rate increased from only 0.01–0.12 mg-N/g-VSS/h during the second incubation. Additionally, the sulfur oxidation rate increased from 0.70 to 1.71 mg-S/g-VSS/h in the second cultivation using ${\text{NO}}_{3}^{-}$ as an electron acceptor, while it decreased under conditions with other electron acceptors (Figure 1B). The concentrations of ${\text{NO}}_{3}^{-}$, ${\text{NO}}_{2}^{-}$, reduced sulfur compounds, and N_2_O measured during the serial subculture process are shown in Supplementary Figure S2. Notably, in the second cultivation, the SADN process was not completed during the test period with electron acceptors other than ${\text{NO}}_{3}^{-}$.

Regarding N_2_O dynamics, the ${\text{NO}}_{3}^{-}$ group exhibited the lowest N_2_O accumulation (Figure 1C) and consumption rates (Figure 1D) during the second subculture (0.007 and 0.006 mg-N/g-VSS/h, respectively). The low but comparable rates suggested a lower N_2_O accumulation capacity when ${\text{NO}}_{3}^{-}$ served as the electron acceptor. In contrast, the NO groups displayed the highest rates of both N_2_O accumulation and consumption rates (1.26 and 0.21 mg-N/g-VSS/h, respectively) among the applied electron acceptors. When N_2_O was supplied as the sole electron acceptor, no further N_2_O accumulation was observed. The increase in the N_2_O consumption rate from 0.01 to 0.12 mg-N/g-VSS/h indicates that enrichment enhanced the N_2_O-reducing capacity via SADN. The net N_2_O accumulation was the lowest in the ${\text{NO}}_{3}^{-}$ group, as shown in Figure 1E. Meanwhile, the N_2_O accumulation rates in the runs with ${\text{NO}}_{2}^{-}$ and NO overwhelmed the corresponding N_2_O consumption rates, resulting in the higher net N_2_O accumulation rates. Together, the ${\text{NO}}_{3}^{-}$ group achieved the highest rates of denitrification and sulfur oxidation during the second subculture with the lowest net N_2_O accumulation, highlighting its potential as a favorable condition for minimizing N_2_O emissions in SADN systems.

### 3.2 Microbial community composition and diversity analysis

**Figure 2:**
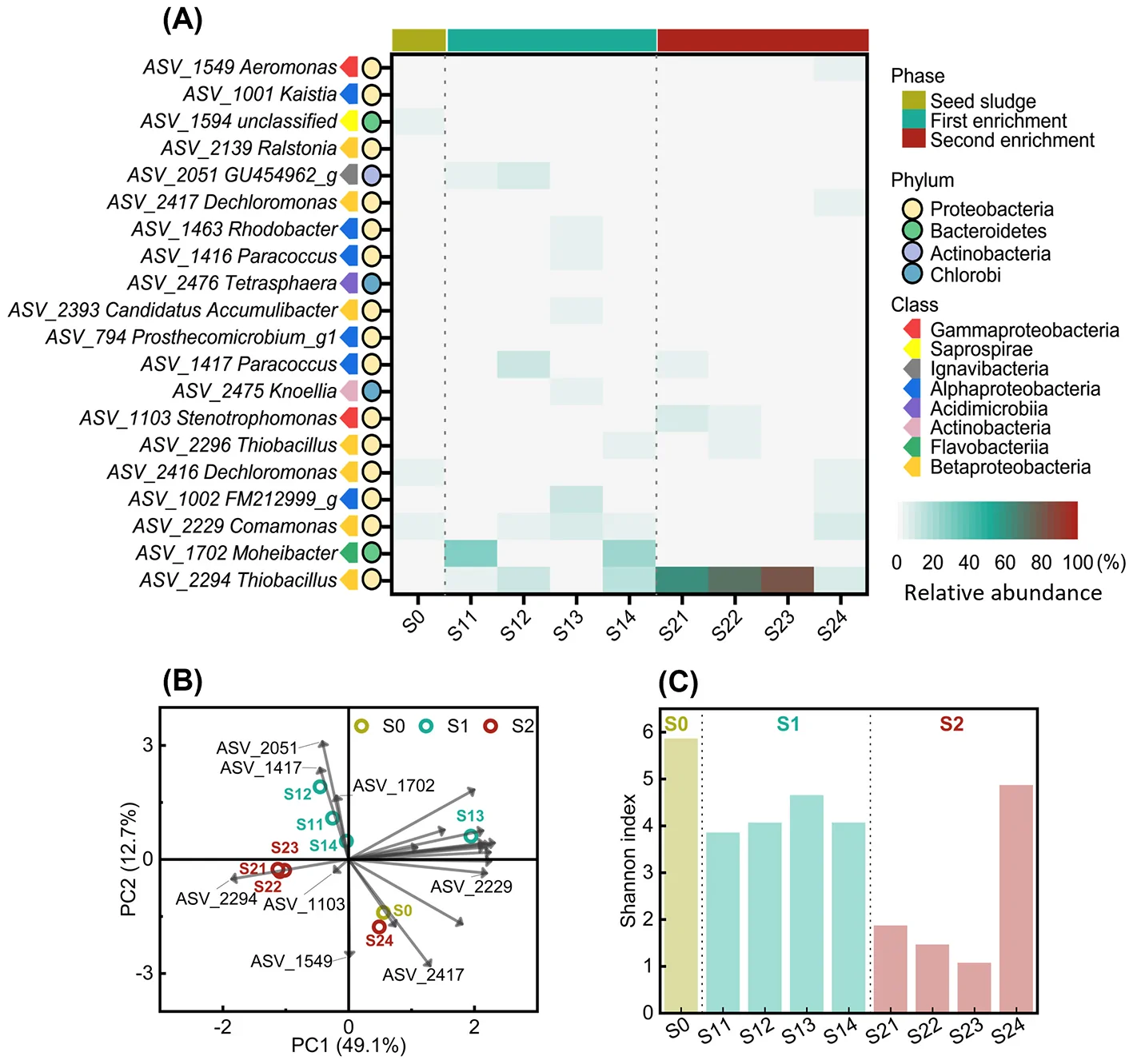
**(A)** Heatmap of the top 20 predominant microbes at the genus level. **(B)** β-diversity through principal component analysis (PCA) to investigate the correlation between enrichment stages (circles) and microbial community structure (gray arrows). **(C)** The Shannon index (α-diversity). S0, S1, and S2 represent seed sludge, the first subculture, and the second subculture with different electron acceptors: ${\text{NO}}_{3}^{-}$ for S11 and S21, ${\text{NO}}_{2}^{-}$ for S12 and S22, NO for S13 and S23, and N_2_O for S14 and S24.

We conducted a comprehensive analysis of microbial communities fed with different electron acceptors using the 16S rRNA gene amplicon sequencing from two sequential subcultures. All sequences across the complete dataset were clustered into 564 unique genera. The relative abundances of the top 20 genera and microbial community diversity, from the inoculum to the second subculture, are shown in Figure 2A. Among these top 20 genera, two *Thiobacillus* amplicon sequence variances (ASVs), namely, ASV_2294 and ASV_2296, were identified. Notably, ASV_2294 *Thiobacillus* was the most dominant ASV in the community and will henceforth be referred to simply as *Thiobacillus*. However, the resolution of 16S rRNA amplicon sequencing precludes discrimination among species within the same genus; species-level characterization of *Thiobacillus* will be further addressed in the metagenomic analysis.

*Thiobacillus* remained dominant throughout the entire enrichment process, with its relative abundance increasing markedly from the first (S1) to the second (S2) subculture (Figure 2), from 6.3% (S11) to 65.8% (S21) in the ${\text{NO}}_{3}^{-}$-fed biomass, from 11.2% (S12) to 75.5% (S22) in the ${\text{NO}}_{2}^{-}$-fed biomass, and from 0.1% (S13) to 84.0% (S23) in the NO-fed biomass. In contrast, the relative abundances of *Moheibacter* and *Comamonas* in these biomasses consistently decreased during the second subculture. With N_2_O as the sole electron acceptor, *Thiobacillus* abundance increased to 13.3% in the first subculture (S14) but subsequently declined to 8.7% in the second (S24). Additionally, distinct community compositions were observed between N_2_O and other electron acceptors at both the phylum and class levels. Detailed information on community compositions at the phylum, class, and genus levels is provided in Supplementary Figure S4.

Figure 2B illustrates the differences in bacterial community structures at the genus level under different electron acceptor conditions and subculture stages, as determined by PCA. A clear differentiation based on the top 20 most abundant ASVs was observed between the first (S1) and second (S2) subcultures. Notably, samples S13 and S24 were distinct from the others along the principal component axes in their respective subculture stages, indicating a unique community shift under NO-fed and N_2_O-fed conditions. Among the top 20 genera, *Stenotrophomonas* was the only genus that showed a positive correlation with *Thiobacillus*, whereas the relative abundances of *Comamonas* and *Dechloromonas* exhibited inverse correlations with that of *Thiobacillus*.

Figure 2C presents the bacterial alpha diversity across all samples. The lowest bacterial diversity (Shannon index of 1.07) was observed under the NO-fed condition in the second subculture, consistent with the strong selective pressure exerted by NO on the microbial community. Except for the N_2_O-fed condition, Shannon indices in the first subculture were higher than those in the second subculture, reflecting the progressive enrichment and convergence of community structure over successive transfers. Although a statistical test was infeasible (*n* = 1), the supplies of ${\text{NO}}_{3}^{-}$, ${\text{NO}}_{2}^{-}$, and NO likely promoted the dominance of *Thiobacillus* and converged the community structures toward lower diversity.

### 3.3 The gene copy numbers of *nir* and *nosZ* during enrichment

**Figure 3:**
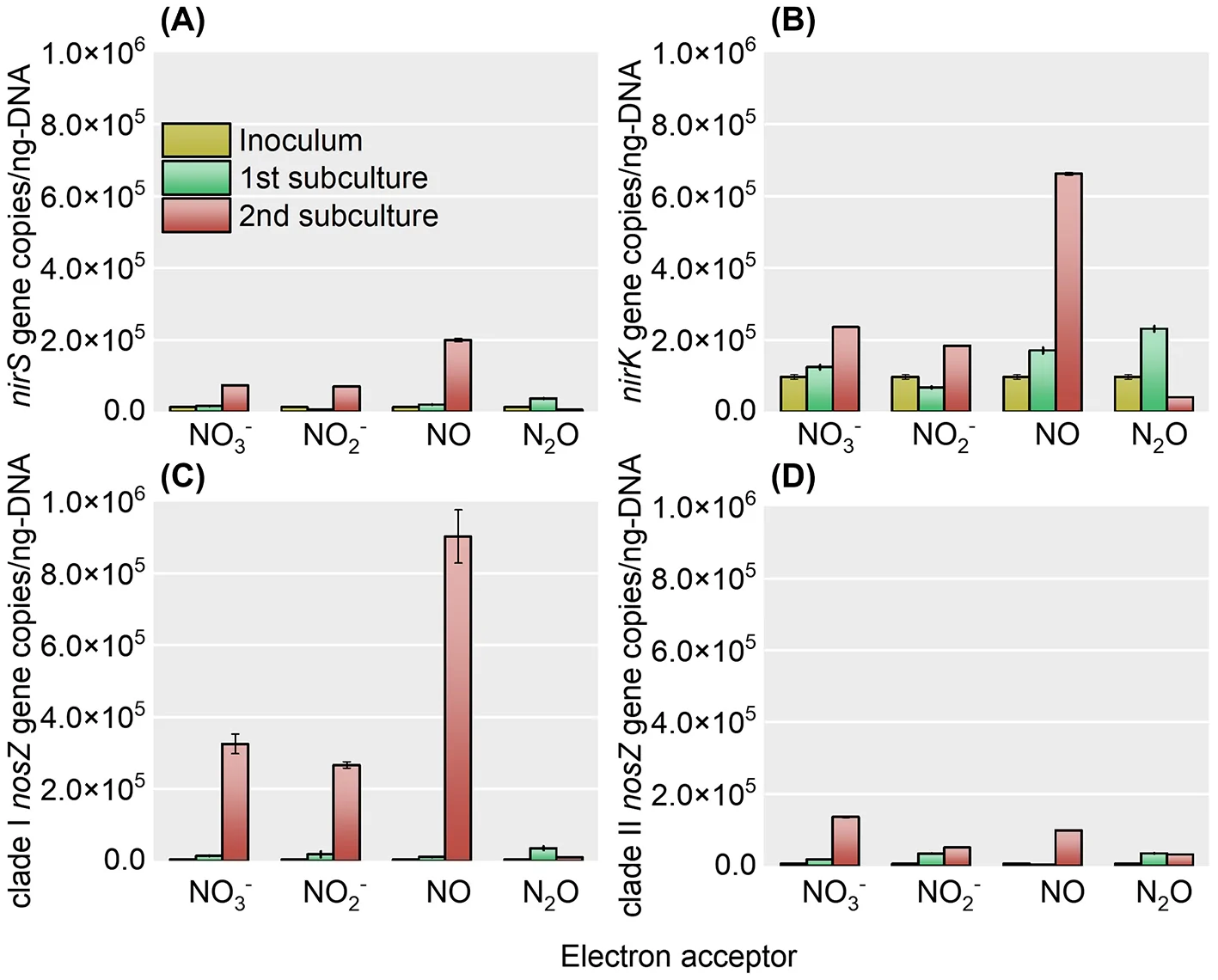
Functional gene copy numbers of **(A)** *nirS*, **(B)** *nirK*, **(C)** clade I *nosZ*, and **(D)** clade II *nosZ* normalized by DNA weight across different electron acceptors and subculture conditions.

Based on the copy numbers of *nirS, nirK*, clade I *nosZ*, and clade II *nosZ* genes under four different electron acceptor conditions (Figure 3), increases in *nirS* and clade I *nosZ* gene copy numbers, by factors of 4.10–5.30 and 3.58–5.96, respectively, were observed during the second subculture, particularly under NO-fed conditions. In contrast, changes in *nirK* gene abundance were marginal, by factors of 4.98–5.37 and 4.98–5.26 under ${\text{NO}}_{3}^{-}$-fed and ${\text{NO}}_{2}^{-}$-fed conditions, respectively. Regarding the relative abundances of *nirS* and *nirK* genes in the second subculture compared to the inoculum, the increase in *nirS* gene was more pronounced than that in *nirK* gene, indicating that *nirS*-type denitrifiers were selectively enriched during prolonged cultivation, especially in the presence of ${\text{NO}}_{3}^{-}$ and ${\text{NO}}_{2}^{-}$ as electron acceptors.

As shown in Figure 3C, the clade I *nosZ* gene encoding the canonical N_2_O reductase exhibited the most significant increase in copy number during the second subculture, particularly under the NO-fed condition. This indicated a more substantial enrichment of clade I *nosZ* microbes during cultivation. The clade II *nosZ* gene also showed an increasing trend, albeit with lower copy numbers compared to clade I *nosZ* (Figure 3D).

### 3.4 Quantitative relationships between *Thiobacillus* with *nir* and *nosZ* genes

**Figure 4:**
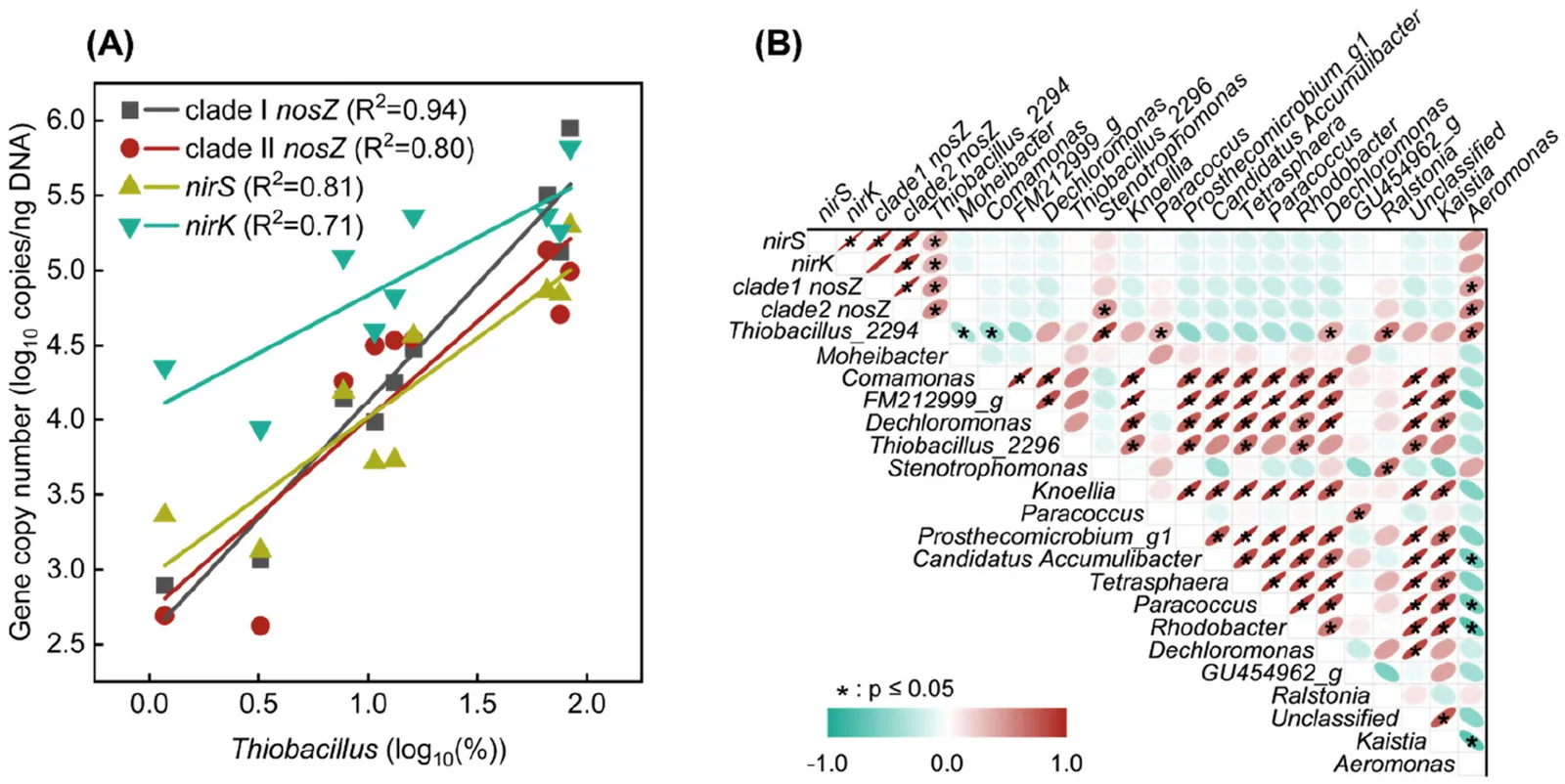
**(A)** Linear regression between logarithmic *Thiobacillus* relative abundance and gene copy numbers of *nirS, nirK*, clade I *nosZ*, and clade II *nosZ*. **(B)** Spearman's rank correlation analysis between the top 20 genus abundance and functional gene copy numbers (*p* ≤ 0.05).

To further elucidate the functional contributions of *Thiobacillus* during enrichment, log-log linear regression analysis was performed between the log10-transformed relative abundance of *Thiobacillus* and the log10-transformed copy numbers of denitrifying genes (Figure 4A). A strong positive correlation was observed between *Thiobacillus* relative abundance and clade I *nosZ* gene copy numbers (*R*^2^ = 0.94). In contrast, clade II *nosZ* displayed a weaker correlation with *Thiobacillus* abundance (*R*^2^ = 0.80), indicating that clade II *nosZ* bacteria belong to other taxa, such as *Dechloromonas, Paracoccus*, or *Stenotrophomonas*, as suggested by the microbial community analysis (Figure 2). Similarly, the *nirS* gene copy numbers showed a positive correlation with *Thiobacillus* relative abundance (*R*^2^ = 0.81), whereas *nirK* gene copy numbers showed only a weak positive correlation (*R*^2^ = 0.71).

As shown in Figure 4B, a complementary Spearman rank correlation heatmap further revealed statistically significant associations (*p* ≤ 0.05) between dominant microbial genera and key denitrification genes. *Thiobacillus* showed positive correlations with *Paracoccus, Dechloromonas*, and *Stenotrophomonas*, all of which were identified as potential denitrifiers in SADN systems. The abundances of *Stenotrophomonas* and *Thiobacillus* were positively correlated with both clade I and clade II *nosZ* gene abundances, indicating their potential contribution to N_2_O consumption across both *nosZ* types. Despite the absence of external organic carbon in the subcultures, *Moheibacter* increased in abundance during the first subculture (Figure 2A) and showed a negative correlation with *Thiobacillus* (Figure 4B).

### 3.5 Metagenome community shifts under different denitrification conditions

A total of 76 high-quality MAGs were recovered from the second-round enrichment cultures. Among these, three MAGs were taxonomically assigned to the genus *Thiobacillus*: MAG 10 (*Thiobacillus* sp. 001899775), MAG 11 (*Thiobacillus* sp. nov.), and MAG 25 (*Thiobacillus thioparus*; Supplementary Table S4). MAG 10 was the most abundant organism (completeness/contamination: 80.3%/0.56%), accounting for 51.1%, 56.4%, and 58.7% of the relative abundances under ${\text{NO}}_{3}^{-}$, ${\text{NO}}_{2}^{-}$, and NO conditions, respectively. In contrast, its relative abundance was only 1.0% under the N_2_O condition (Supplementary Table S5). This pattern is consistent with the 16S rRNA amplicon sequencing results (Figure 2A). MAG 11 (completeness/contamination: 84.03%/0.20%) reached a relative abundance of 1.2% under the ${\text{NO}}_{3}^{-}$ condition but remained below 0.1% in the remaining three conditions. Unlike MAG 10 and MAG 11, MAG 25 (completeness/contamination: 77.2%/0.75%) exhibited its highest relative abundance of 1.8% specifically under the N_2_O condition.

Notably, the average nucleotide identity (ANI) of MAG 11 was 72.6%, well below the commonly accepted species-level threshold of 95%, suggesting that MAG 11 likely represents a previously undescribed species (Supplementary Table S4). Phylogenetic analysis showed that MAG 11 clustered closely with MAG 10, while MAG 25 formed an adjacent clade (Supplementary Figure S5). Collectively, these genomic and phylogenetic data support the conclusion that MAG 11 represents a novel species within the genus *Thiobacillus*, distinct from previously characterized members.

Beyond *Thiobacillus*, the sulfur-oxidizing bacterium MAG 38 (*Sulfuricystis*) became the dominant autotroph (4.5%) under the N_2_O condition, while MAG 07 (*Azonexus* sp. 040275885), a dedicated N_2_O-reducing bacterium, was detected at 1.2% relative abundance (Supplementary Table S5). The community under the N_2_O condition (S24) was further characterized by a higher proportion of heterotrophic microorganisms, including MAG 06 (*Phosphoribacter*, 2.07%) and MAG 27 (*Ottowia*, 2.19%). Taken together, the ${\text{NO}}_{3}^{-}$, ${\text{NO}}_{2}^{-}$, and NO conditions consistently supported a community dominated by the chemolithoautotrophic sulfur-oxidizer *Thiobacillus* sp. 001899775, whereas the N_2_O condition fostered a more functionally diverse community comprising both autotrophic sulfur oxidizers, e.g., *Thiobacillus thioparus* and *Sulfuricystis*, and heterotrophic taxa.

### 3.6 Differential functional gene and metabolic pathways among key MAGs

**Figure 5:**
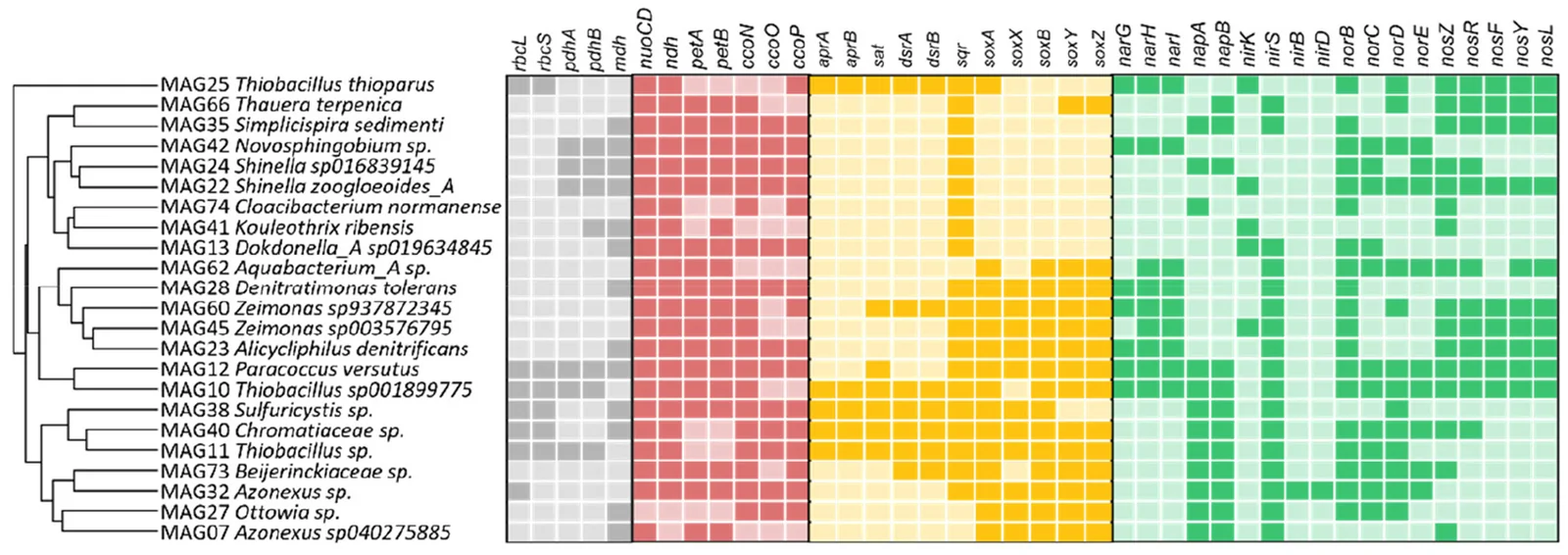
Metabolic potential and genomic functional distribution of key metagenome-assembled genomes (MAGs). The heatmap illustrates the presence and absence of key functional genes involved in carbon fixation (gray), electron transport (red), sulfur oxidation (yellow), and denitrification (green). Dark-filled blocks indicate the presence of the corresponding functional ortholog within each MAG; light-shaded blocks indicate absence.

To characterize the functional potential of the recovered MAGs, functional annotation was performed on 23 MAGs encoding genes associated with sulfur oxidation or denitrification (Figure 5). It should be noted that the completeness of MAG11 (84.0%) and MAG25 (77.2%) falls below the conventional threshold for high-quality genomes (completeness ≥ 90%). The apparent absence of specific functional genes in these MAGs may therefore reflect incomplete genome assembly or limitations of the binning process, rather than the true absence of those genes from the organism's genome. The absence of these genes should not be taken as definitive evidence.

Among the three MAGs [MAG 07 (*Azonexus* sp. 040275885), MAG 41 (*Kouleothrix ribensis*), and MAG 66 (*Thauera terpenica*)] shared the genomic signature of carrying *nosZ* in the partial (MAG 07 and MAG 41) or entire (MAG 66) absence of NO reduction genes (Figure 5), indicating the metabolic potential to reduce N_2_O to N_2_ but not to reduce NO. MAG 13 (*Dokdonella* sp.), MAG 27 (*Ottowia* sp.), MAG 28 (*Denitratimonas tolerans*), MAG 32 (*Azonexus* sp.), and MAG 042 (*Novosphingobium* sp.) harbor the *norB*/*norC* genes but lack the *nosZ* gene, suggesting that these organisms possess the genetic potential to produce N_2_O and likely terminate denitrification at N_2_O.

**Figure 6:**
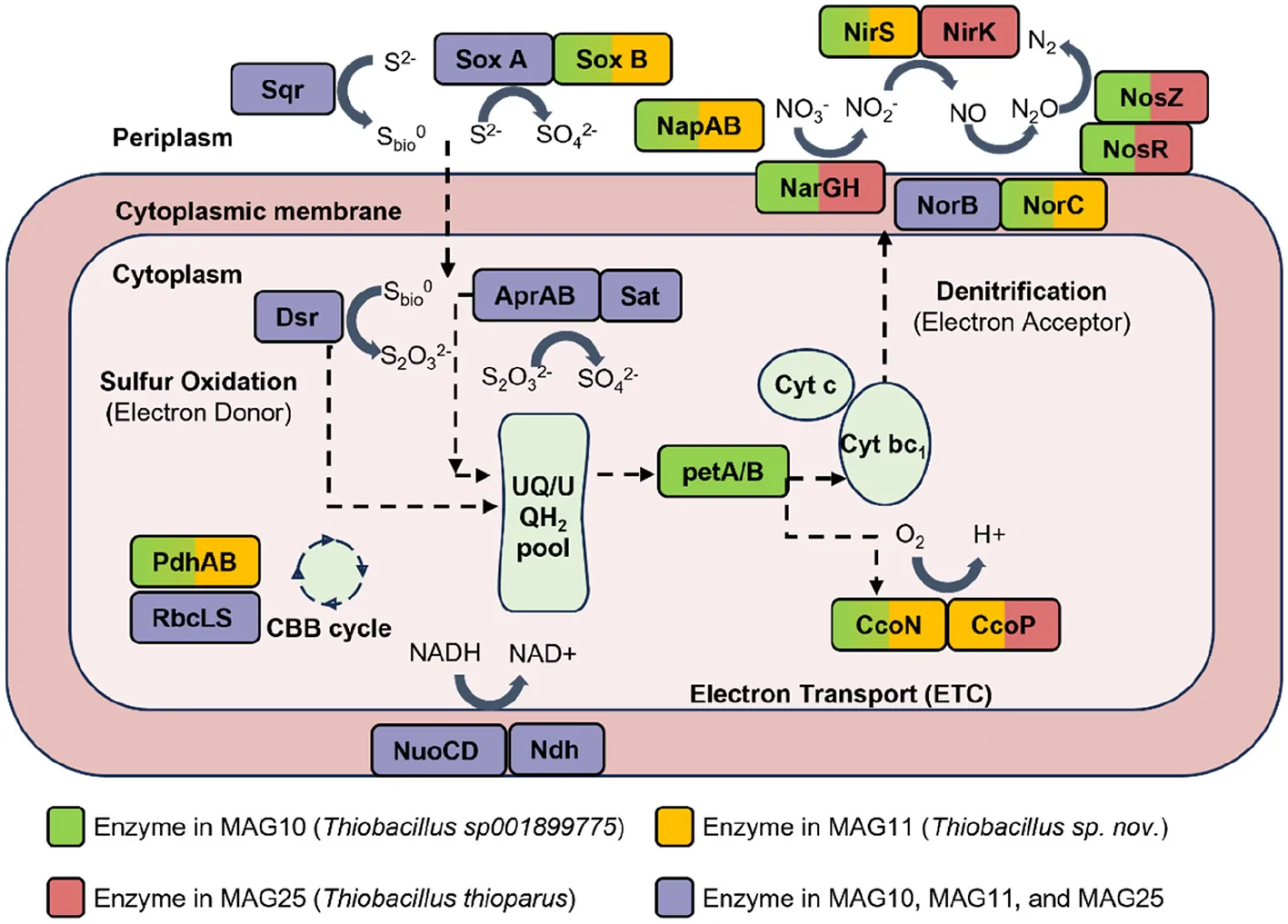
Proposed metabolic framework and functional gene distribution of three *Thiobacillus* MAGs.

Functional gene annotation further revealed that the three *Thiobacillus* MAGs [MAG 10 (*Thiobacillus* sp. 001899775), MAG 11 (*Thiobacillus* sp.), and MAG 25 (*Thiobacillus thioparus*)] differ not only in their nitrogen reduction potentials but also in their sulfur oxidation gene repertoire, carbon fixation machinery, and electron transport chain composition (Figure 6 and Supplementary Table S6). MAG 10 encodes a complete suite of sulfur oxidation genes (*sqr*-*dsr*-*apr*-*sat*-*sox*), indicating the potential for the sequential oxidation of sulfide to elemental sulfur, sulfite, and ultimately to sulfate. In addition, MAG 10 harbors a complete denitrification pathway (*nap*-*nirS*-*norB*-clade I *nosZ*), along with the accessory protein gene *nosR*. It also encodes the cytochrome *bc*_1_ complex (*petAB*) for a robust electron transport capacity. The absence of malate dehydrogenase (*mdh*) and other genes involved in organic carbon assimilation indicates that MAG10 is a strict chemolithoautotroph that relies exclusively on sulfur oxidation coupled to denitrification, with carbon fixation via the Calvin–Benson–Bassham (CBB) via *rbcL* and *rbcS*.

MAG 11 encodes *nap, nirS*, and *norB*, while *nosZ* was not detected within the assembled genome (completeness: 84.0%), consistent with nitrate reduction terminating at N_2_O. In contrast, MAG 25 encodes the entire set of denitrification genes. Unlike MAGs 10 and 11, it possesses the *nirK* gene, which encodes a copper-containing nitrite reductase. Regarding the electron transport chain, MAG 25 lacks *petAB* and *pdhAB* and encodes only *ccoP* genes. Both MAG 11 and MAG 25 encode *rbcL*/*rbcS* and lack the malate dehydrogenase gene *mdh*, suggesting the metabolic potential of CO_2_ fixation and an incomplete TCA cycle. The presence of *rbcL/rbcS* genes is consistent with a strictly chemolithoautotrophic metabolism.

### 3.7 Differential gene abundance of functional genes across *Thiobacillus* MAGs under varying electron acceptor conditions

**Figure 7:**
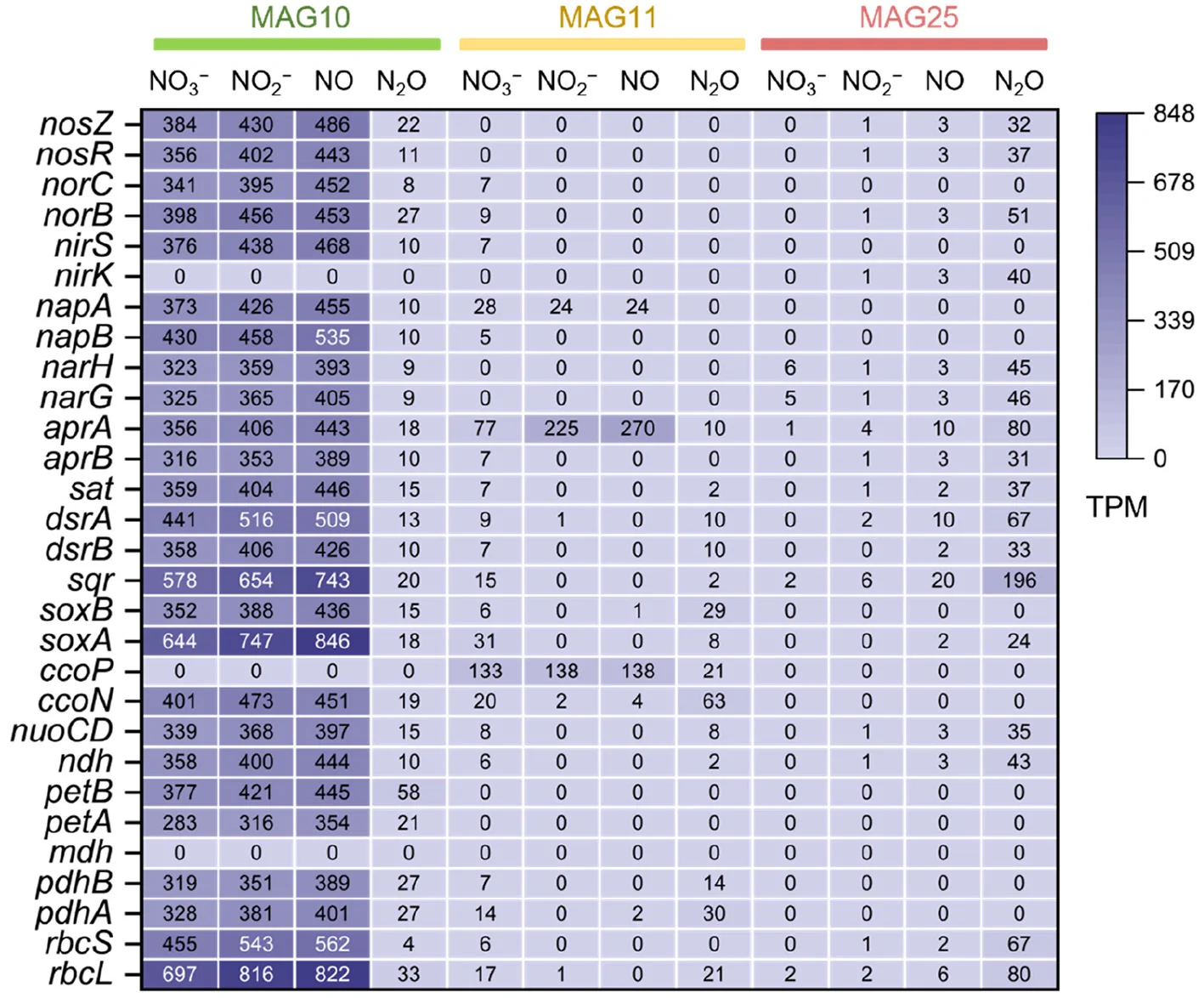
Transcripts per million (TPM) normalized abundances based on mapped metagenomic reads of key functional genes in three *Thiobacillus* MAGs under different nitrogen oxide electron acceptor conditions. S21: ${\text{NO}}_{3}^{-}$, S22: ${\text{NO}}_{2}^{-}$, S23: NO, and S24: N_2_O.

Under four operational conditions defined by distinct nitrogen oxide electron acceptors (S21: ${\text{NO}}_{3}^{-}$, S22: ${\text{NO}}_{2}^{-}$, S23: NO, S24: N_2_O), the functional gene abundance profiles of the three *Thiobacillus* MAGs, quantified by metagenomic reads per million- (TPM-) normalized abundances based on mapped metagenomic reads, revealed condition-dependent divergence in genomic representation (Figure 7).

MAG 10 maintained comprehensively higher TPM value across the ${\text{NO}}_{3}^{-}$, ${\text{NO}}_{2}^{-}$, and NO conditions than N_2_O condition, with sulfur oxidation (*sqr*: 578–743; *soxA*: 644–846; *dsrA*: 441–516), the CBB cycle (*rbcL*: 697–822; *rbcS*: 455–562), electron transport (*petA*: 283–354; *petB*: 377–445; *ccoN*: 401–473), and the denitrification pathway (*nirS*: 376–468; *norB*: 398–456; *norC*: 341–452; *nosZ*: 384–486), respectively (Figure 7). In the N_2_O condition, the abundance of all MAG 10 functional genes declined sharply with TPM values of 27 and 22, respectively.

MAG 11 displayed strikingly low functional gene abundance for most genes across S21-S23, with two notable exceptions. *aprA* exhibited a remarkable increase in abundance under ${\text{NO}}_{2}^{-}$ and NO conditions with TPM values of 225 and 270, while its catalytic partner *aprB* remained at near zero under the same conditions. Under the N_2_O condition, MAG 11 increased the TPM values for *ccoN, pdhA, dsrA*, and *rbcL* genes (63, 30, 10, and 21, respectively). Under all electron-acceptor conditions, MAG 11 rarely showed near-zero TPM abundance for *nirK, nirS, norB, norC*, and *nosZ*, suggesting that it largely lacks the genetic capacity for denitrification under the conditions tested.

MAG 25 exhibited minimal functional gene abundances among three *Thiobacillus* MAGs (MAGs 10, 11, and 25) under ${\text{NO}}_{3}^{-}$, ${\text{NO}}_{2}^{-}$, and NO conditions (S21–S23), with most functional genes detected at near-zero levels. The result indicates that MAG 25-affiliated organisms constituted only a minor fraction of the active community under these conditions. However, in the N_2_O condition (S24), MAG 25 increased the abundances of functional genes across all categories. Collectively, the functional gene abundance profiles of the three *Thiobacillus* MAGs reveal a clear functional stratification.

## 4 Discussion

In this study, the three *Thiobacillus* MAGs exhibited distinct functional roles in N_2_O production and reduction, highlighting the metabolic plasticity of *Thiobacillus* in sulfur-driven autotrophic denitrification systems. The ecological role of *Thiobacillus* appeared to be highly condition-dependent and regulated by environmental and operational factors.

### 4.1 Electron acceptor governs SADN performance and N_2_O emission

The nature of nitrogen oxides as electron acceptors exerted a decisive influence on both denitrification efficiency and N_2_O emission dynamics in the SADN system. Under a ${\text{NO}}_{3}^{-}$-fed condition, the system achieved the highest denitrification and sulfur oxidation rates (2.41 mg-N/g-VSS/h and 1.71 mg-S/g-VSS/h, respectively) while maintaining minimal net N_2_O accumulation (Figure 1). The observed stoichiometric ratio of sulfate production to nitrate reduction (1.4 mg-S/mg-N) aligns closely with the theoretical values established for SADN (Cardoso et al., 2006). In contrast, ${\text{NO}}_{2}^{-}$- and NO-fed conditions markedly decreased sulfur oxidation rates, accompanied by substantial N_2_O accumulation. In particular, the NO-fed condition was striking, with an N_2_O accumulation rate of 1.26 mg-N/g-VSS/h, indicating a severe bottleneck at the N_2_O reduction step. Although the N_2_O-fed condition showed the lowest overall denitrification rates, the progressive increase in N_2_O consumption observed during successive subculturing suggests a shift in the microbial community composition toward more effective N_2_O-consuming populations (Figure 1D).

The mechanistic basis for the N_2_O metabolic divergence lies in the differential effects of each electron acceptor on N_2_O reduction activity. Under ${\text{NO}}_{3}^{-}$-fed conditions, rapid sulfide consumption (Supplementary Figure S2) curtailed the duration of sulfide-mediated NosZ inhibition, while *Thiobacillus* maintained coordinated expression of the *nar-nir-nor-nos* gene cluster (Beller et al., 2006b), suggesting that transiently produced N_2_O was reduced as soon as it formed. In the ${\text{NO}}_{2}^{-}$-fed condition, free nitrous acid likely suppressed *nosZ* transcription even at micromolar concentrations, decoupling the denitrification chain (Yang et al., 2016b; Zhou et al., 2022). In the NO-fed condition, although NO serves as a potent transcriptional inducer of *nosZ* gene, the transient intracellular accumulation of NO at high concentrations may directly inhibit *nosZ* activity (Figure 1C), generating an “electron bottleneck” in which electron flux toward *norB* exceeded the processing capacity of *nosZ*, resulting in pronounced N_2_O production (Spiro, 2012). Collectively, these results demonstrate that the choice of the electron acceptor shapes not only the thermodynamic potential for denitrification but also, critically, the N_2_O emission patterns by regulating both *nosZ* gene expression and enzyme activity.

The consistent dominance of *Thiobacillus* under ${\text{NO}}_{3}^{-}$, ${\text{NO}}_{2}^{-}$, and NO-fed conditions likely reflects a combination of bioenergetic and genomic adaptations that confer a competitive advantage. ${\text{NO}}_{3}^{-}$, ${\text{NO}}_{2}^{-}$, and NO have a larger electron-accepting capacity per mole of nitrogen than N_2_O and serve as more energetically favorable electron acceptors under the tested conditions (Zumft, 1997), thereby promoting the proliferation of *Thiobacillus*. Moreover, the dominant *Thiobacillus* species (MAG 10) possesses a complete sulfur oxidation–denitrification pathway (Section 3.6), through which electrons generated from sulfide oxidation are efficiently transferred via an intracellular electron transport chain to denitrification reductases. The tight coupling of these processes reduces electron loss and likely provides a competitive growth advantage over microorganisms with incomplete denitrification capabilities. *Thiobacillus* could rapidly oxidize and detoxify sulfide (Supplementary Figure S2), thereby protecting its NosZ activity from sulfide-mediated inhibition (Cardoso et al., 2006) and reinforcing its dominance over competitors that are more sensitive to residual sulfide toxicity.

### 4.2 Electron acceptor shapes community succession and *nosZ* clade type

The performance divergence in varying electron acceptors was mirrored by directional shifts in community composition, the central feature of which was the distribution of different *nosZ* clades (clade I and clade II *nosZ* denitrifiers). Under ${\text{NO}}_{3}^{-}$, ${\text{NO}}_{2}^{-}$, and NO-fed conditions, *Thiobacillus*, harboring clade I *nosZ*, consistently dominated the community (Figure 2A), and its relative abundance correlated positively with the copy numbers of *nir* and *nos* functional genes (Figure 4), confirming its role as the primary driver of coupled sulfur oxidation and complete denitrification under these conditions (Jones et al., 2013; Martínez-Santos et al., 2018). The most pronounced increase in clade I *nosZ* gene copy numbers (239-fold increase) occurred in the NO-fed group (Supplementary Figure S6), consistent with the cytotoxic nature of NO, which may have selectively suppressed microorganisms lacking NO-detoxifying capacity, thereby contributing to the observed community shift under NO-fed conditions (Spiro, 2012).

When N_2_O served as the sole electron acceptor, *Thiobacillus* abundance declined to 8.7% (Figure 2A), and the community shifted toward a more diverse assemblage dominated by *Azonexus* and *Dechloromonas*. The mechanistic insights underlying this succession are twofold. As an obligate chemolithoautotroph, *Thiobacillus* could prioritize electron acceptors with higher energy yields (${\text{NO}}_{3}^{-}$ > ${\text{NO}}_{2}^{-}$ > NO > N_2_O) to sustain the energetic demands of carbon fixation via the CBB cycle (Hudson, 2024). Second, clade I *nosZ* gene expression in canonical denitrifiers requires induction by upstream intermediates such as NO or ${\text{NO}}_{2}^{-}$ (Conthe et al., 2018). In an N_2_O-fed condition, the absence of gene induction deterred the predominance of *Thiobacillus*. The *Azonexus* (MAG 07), which carries a clade II *nosZ* gene with significantly higher substrate affinity for N_2_O and a more efficient electron transport system (Sanford et al., 2012; Hallin et al., 2018), was likely to thrive in N_2_O-fed conditions, offering a potential selective advantage when N_2_O is the only available electron acceptor. It is important to emphasize that even under *Thiobacillus*-dominated conditions, effective N_2_O consumption depended on synergistic interactions with clade II *nosZ* denitrifiers, since the gene copy number of clade II *nosZ* also increased after enrichment cultivation (Figure 4).

Electron acceptors could contribute to the fundamentally distinct biochemical and ecological properties of the two *nosZ* clades. *Thiobacillus* (clade I *nosZ*) uses the twin-arginine translocation (Tat) system for periplasmic export, embedded within complete denitrification gene clusters (Zumft, 1997; Sanford et al., 2012). In contrast, clade II *nosZ* denitrifiers employ the Sec secretion pathway, are commonly found in non-denitrifying microorganisms that lack *nir* and/or *nor* genes. Moreover, they display substantially higher affinity for N_2_O, with apparent N_2_O affinity constants reported to be one to two orders of magnitude lower than those of clade I *nosZ* N_2_O bacteria (Hallin et al., 2018; Conthe et al., 2019). These kinetic differences mean that clade II *nosZ* bacteria can effectively scavenge trace N_2_O that remains thermodynamically inaccessible to clade I *nosZ* bacteria, making them disproportionately important for N_2_O mitigation at low N_2_O partial pressures (Qi et al., 2022). The co-enrichment of both clade types therefore represents a functionally complementary N_2_O reduction network: clade I *nosZ* bacteria, i.e., *Thiobacillus*, reduce N_2_O under high-flux denitrification conditions, while the coexisting clade II *nosZ* bacteria, showing high N_2_O affinity, utilize the residual N_2_O. Therefore, the enrichment of sulfur-oxidizing bacteria alone cannot fully explain the N_2_O-reduction capacity in SADN. Profound consideration should be given to maintaining the balance between clade I and clade II *nosZ*-containing populations, as well as the integrity of the microbial symbiotic network.

### 4.3 Electron acceptors lead diverse *Thiobacillus* functional modules

While *Thiobacillus* is traditionally viewed as a monolithic functional group in SADN systems, our metagenomic analysis reveals functional modularity within a genus (Figure 5). Despite their phylogenetic proximity, MAG 11 and MAG 25 exhibit distinct genomic architectures that reflect divergent ecological strategies under varying electron acceptor pressures. At the genomic level, MAG 11 encoded not only an incomplete denitrification pathway, but also exhibited near-zero relative abundances of the *nar, nir, nor*, and *nos* genes under all denitrifying conditions. These results suggest that MAG 11 functioned primarily as a sulfur-oxidizing autotroph rather than an active denitrifier in this microbial community. It is essential to understand that gene abundance and transcript levels do not directly correspond to enzyme activity or metabolic rates. Therefore, although our results suggest a potential involvement in N_2_O metabolism, validating the functional significance requires direct measurements of enzymatic activity and phenotypic assays, which should be explored in future studies. Within its sulfur oxidation process and electron transfer, MAG11 exhibits two condition-specific anomalies that collectively suggest a biochemically distinct strategy relative to MAG 10. MAG 11 exhibits condition-specific anomalies in *aprA* gene abundance under ${\text{NO}}_{2}^{-}$ and NO conditions, indicating a preferential reliance on the AprAB-Sat pathway over the Sox system for sulfite oxidation (Ghosh and Dam, 2009). Moreover, the persistent high abundance of *ccoP* (encoding *cbb*_3_-type cytochrome *c* oxidase) in the absence of its catalytic partner *ccoN* (encoding the catalytic subunit I) is equally anomalous (Figure 7). However, these anomalous results are likely attributable to limitations in the genome assembly or binning process. MAG11 exhibited condition-specific abundance of the *aprA* (encoding the alpha subunit of adenosine5′-phosphosulfate reductase, AprA) and *ccoP* genes, although the mechanism underlying this pattern remains unclear and warrants further investigation (Beller et al., 2006b; Roothans et al., 2025).

The co-existence of *sqr* (encoding sulfide: quinone oxidoreductase, SQR) and *nirK* genes in MAG 25 in the N_2_O-fed condition is not coincidental but reflects a mechanistically coherent electron transport mechanism rooted in the bioenergetics of chemolithoautotrophic sulfur oxidation (Figure 6). SQR serves as the membrane-bound entry point of sulfur oxidation, feeding electrons into the bacterial electron transport chain to NirK in the periplasm, which acts as a copper-containing nitrite reductase (Hell, 2008). The high *sqr* TPM values observed in MAG 25 exclusively under N_2_O conditions (Figure 7) suggest that sulfide oxidation of MAG 25 (*Thiobacillus thioparus*) is selectively activated to generate the reductant flux required to support NosZ-mediated N_2_O respiration. NirS is typically induced by ${\text{NO}}_{2}^{-}$ and NO through nitric oxide and nitrate reductase regulator (NNR)-type regulators. Conversely, NirK may respond more directly to the redox state of the periplasm, which is linked to its copper-center structure and specific electron donor preferences (Zumft, 1997; Rinaldo et al., 2008), potentially giving *nirK*-containing denitrifiers a metabolic advantage. Hence, the higher *sqr* abundance and the regulatory advantage of *nirK* under N_2_O-fed conditions collectively suggest that MAG 25 integrates sulfur oxidation with terminal denitrification via the SQR–menaquinone–PetAB–NirK–NosZ relay specifically when N_2_O is the dominant electron acceptor.

### 4.4 Condition-dependent dual roles of *Thiobacillus* in N_2_O accumulation and consumption

Under ${\text{NO}}_{2}^{-}$ and NO conditions, *Thiobacillus* tended to function as a net N_2_O producer since the NO-fed group exhibited the highest net N_2_O accumulation among all tested conditions (Figure 1E). Studies have shown that electron-donor limitations and ${\text{NO}}_{2}^{-}$ buildup can lead to incomplete denitrification and increased N_2_O accumulation in SADN. This suggests that inadequate electron supply, ${\text{NO}}_{2}^{-}$ stress, or low *nosZ* expression may cause the dominant full-fledged denitrifying *Thiobacillus* (MAG 10, Figure 6) to favor incomplete denitrification, leading to net N_2_O production. Furthermore, the incomplete denitrifying *Thiobacillus* (MAG 11, Figure 6) may act as an N_2_O producer during specific operational stages (3.1).

In contrast, *Thiobacillus* can also function as an efficient N_2_O consumer under ${\text{NO}}_{3}^{-}$ and N_2_O conditions. At the MAG level, MAG 10 (the dominant *Thiobacillus*) microorganism across ${\text{NO}}_{3}^{-}$, ${\text{NO}}_{2}^{-}$, and NO conditions maintained stable *nosZ* gene abundance (TPM: 384–487) across the first three conditions, and its *norB* and *nosZ* genes were relatively better retained than other functional genes under the N_2_O condition (TPM: 27-22), consistent with its role as the primary N_2_O sink within the *Thiobacillus* community (Figure 7). Physiological studies have confirmed that *Thiobacillus denitrificans* can grow chemoautotrophically using N_2_O as a terminal electron acceptor, thereby coupling sulfur oxidation with N_2_O reduction (Beller et al., 2006a; Kelly and Wood, 2000b). Moreover, several studies have demonstrated that SADN systems possess the intrinsic potential to mitigate N_2_O accumulation by stimulating *nosZ* expression and enhancing N_2_O-reducing activity (Yang et al., 2016b; Cao et al., 2021; Wang et al., 2023). The present study supported the enrichment of MAG 10 under N_2_O-fed conditions, suggesting its potential contribution as an N_2_O sink within an SADN reactor.

Collectively, these findings indicate that *Thiobacillus* should not be simply categorized as either an N_2_O producer or consumer in SADN systems. Instead, its functional role appears to be dynamically regulated by environmental conditions, particularly sulfur availability, ${\text{NO}}_{2}^{-}$ accumulation, and the balance between N_2_O-producing and N_2_O-reducing activities. Therefore, regulating operational parameters could convert *Thiobacillus* populations from significant sources of N_2_O into efficient sinks, with notable implications for reducing N_2_O emissions in SADN systems.

This study also offers direct practical insights for designing and operating continuous SADN systems. The distinct hierarchy of N_2_O emission levels across electron-acceptor conditions (${\text{NO}}_{3}^{-}$ < ${\text{NO}}_{2}^{-}$ < NO) provides a mechanistic basis for selecting appropriate electron acceptors in engineered setups. Prioritizing ${\text{NO}}_{3}^{-}$ as the primary electron acceptor while minimizing ${\text{NO}}_{2}^{-}$ and NO buildup likely enhances the proliferation of *Thiobacillus* (from 6.3% to 65.8%) and reduces N_2_O emissions, facilitated by the development of a microbial community. This approach can shorten the startup phase of continuous-flow SADN reactors. Additionally, the N_2_O emission data across the four tested electron-acceptor conditions serve as references for predicting emission patterns in full-scale SADN reactors during periods of performance instability. Finally, the community deterioration observed in the third subculture, which is attributable primarily to a progressive decline in biomass concentration resulting from repeated 20% (v/v) transfers under strict inorganic, organic-carbon-free conditions. It underscores the importance of maintaining sufficient biomass levels in continuous SADN systems. In addition, maintaining dissolved oxygen at sufficiently low but stable levels remains essential to sustain anaerobic sulfur oxidation and avoid sulfide inhibition of N_2_O reduction activity during full-scale implementation (Cardoso et al., 2006), although direct DO measurements under the third-subculture conditions were not performed.

## 5 Conclusion

This study demonstrated that nitrogen oxide electron acceptors fundamentally regulate denitrification performance, microbial community succession, and N_2_O dynamics in sulfur-driven autotrophic denitrification systems. ${\text{NO}}_{3}^{-}$ supported efficient sulfur oxidation and complete denitrification with minimal N_2_O accumulation, whereas ${\text{NO}}_{2}^{-}$ and NO promoted incomplete denitrification and elevated N_2_O emissions. Distinct enrichment patterns of clade I and clade II *nosZ* bacteria revealed complementary ecological roles in N_2_O reduction. Genome-resolved analyses further demonstrated substantial functional differentiation within *Thiobacillus* populations, including condition-dependent shifts between N_2_O production and consumption. Genome-resolved analyses revealed that *Thiobacillus* with different denitrifying genotypes could act as either a potential N_2_O sink or source, depending on the types of electron acceptor. These findings highlight the metabolic plasticity of *Thiobacillus* and emphasize that effective mitigation of N_2_O emissions in SADN systems depends not only on enrichment of sulfur-oxidizing bacteria but also on maintaining balanced *nosZ* clade interactions and stable microbial symbiotic networks.

## Data Availability

The datasets presented in this study can be found in online repositories. The names of the repository/repositories and accession number(s) can be found in the article/Supplementary material.
