# Supplementary material for: Electron acceptor-dependent duality of nitrous oxide metabolism in Thiobacillus during sulfur autotrophic denitrification
Source: Front Microbiol. 2026 Sep 4;17:1903473. doi: 10.3389/fmicb.2026.1903473 (PMC13585965; doi:10.3389/fmicb.2026.1903473)
Supplement: Supplementary file 1 [file Data_Sheet_1.docx]

**[Supplementary information]**

**Electron acceptor-dependent duality of nitrous oxide metabolism in *Thiobacillus* during sulfur autotrophic denitrification**

**Running title: NOx-driven N_2_O metabolism by *Thiobacillus***

**Muchun Zhou^1^, Shohei Yasuda^2^, Hiroto Miura^1^, Tianxiang Xu^1^, Megumi Kuroiwa^1^, Xiaoguang Xu^3^, Akihiko Terada^1,^***

^1^ Department of Applied Physics and Chemical Engineering, Tokyo University of Agriculture and Technology, Tokyo, 184-8588, Japan

^2^ Civil Engineering, School of Engineering, College of Science and Engineering, University of Galway, Galway, H91 TK33, Ireland

^3^ School of Environment, Nanjing Normal University, Nanjing, 210023, China

***Correspondence:** Akihiko Terada

**E-mail address:** [akte@cc.tuat.ac.jp](mailto:xxg05504118@163.com)

**Keywords:** Sulfur autotrophic denitrification; electron acceptor; nitrous oxide; *Thiobacillus*; clade I */* II *nosZ* gene

1. **Supplementary Data**

**1.1** **Calculation of the biomass-specific denitrification rate and sulfur oxidation rate**

A half-order reaction model based on the Monod equation has been widely applied to calculate the kinetics of denitrification and sulfur oxidation rates for sulfide-based autotrophic denitrification (Koenig, 2001; Xu et al., 2014). And the relevant chemical reaction equations are shown below (Lin et al., 2018; Oberoi et al., 2021):

$$R_{{NO}_{X}}=-\frac{\Delta C_{{NO}_{X},t}}{\Delta t\times VSS}$$

$$R_{{{SO}_{4}}^{2-}}=\frac{\Delta C_{{{SO}_{4}}^{2-}, t}}{\Delta t\times VSS}$$

where,$R_{{NO}_{x}}$comprised of $R_{{{NO}_{3}}^{-}},R_{{{NO}_{2}}^{-}}{, R}_{NO}, R_{N_{2}O}$(mg-N/g-VSS/h), and $R_{{{SO}_{4}}^{2-}}$ (mg-S/g-VSS/h) are the reaction rates measured by the experiment under the electron acceptor condition of NO_3_^−^, NO_2_^−^, NO, and N_2_O, respectively. $R_{{NO}_{x}}$ and $R_{{{SO}_{4}}^{2-}}$ were determined using linear regression of the linear part of each concentration profile during the period with the highest linearity. $R_{{{NO}_{3}}^{-}}, R_{{{NO}_{2}}^{-}}{, R}_{NO}, R_{N_{2}O}$, and $R_{{{SO}_{4}}^{2-}}$ are true specific reaction rates of NO_3_^−^, NO_2_^−^, NO, N_2_O, and SO_4_^2−^. $\Delta C_{{NO}_{X},t}$ (mg-N/L) and $\Delta C_{{{SO}_{4}}^{2-}, t}$ (mg-S/L) are the concentration changes in sulfate and nitrous compounds over time. Due to the high cell toxicity of NO, some denitrifying bacteria possess a strict regulatory system to curb intracellular NO accumulation, and the accumulated NO can be marginal (Oberoi et al., 2021). When NO detoxification occurs, the end product should be N_2_O. Nevertheless, the amount of accumulated N_2_O (Figure S2F) does not explain the material balance. An alternative biochemical or chemical reaction should occur. Because NO is highly reactive, the following chemical reaction likely occurred.

2NO + H_2_O + 0.5O_2_ → 2HNO_2_

The assumption is plausible based on Figures S2E and S2F, in which NO_2_^-^ but not NO was detected at the onset of the batch experiment. In addition, the NO_2_^-^ concentration was comparable to the theoretical NO concentration at the beginning (82 mg-N/L). It should be noted that although oxygen removal was achieved in this study, a trace amount of oxygen remained, driving HNO_2_ (HNO_2_ ↔ NO_2_^-^ + H^+^) production from NO. This means the end product of the supplemented NO was HNO_2_/NO_2_^-^, but not N_2_O. Given this point, the true reduction rate of NO was assumed as follows:

$$R_{NO}=R_{{{NO}_{2}}^{-}}$$

**1.2. Calculation of the N_2_O accumulation rate and consumption rate**

The rates of N_2_O accumulation and consumption were determined from the experimentally measured N_2_O concentrations in the headspace. Under conditions where N_2_O acted as the sole electron acceptor, the N_2_O consumption rate was considered equivalent to the overall denitrification rate.

$$R_{N_{2}O,a}=\frac{\Delta C_{N_{2}O,a}}{\Delta t\times VSS}$$

$$R_{N_{2}O,c}=-\frac{\Delta C_{N_{2}O,c}}{\Delta t\times VSS}$$

$$R_{N_{2}O,net}=R_{N_{2}O,a}{-R}_{N_{2}O,c}$$

where$R_{N_{2}O,a}$, $R_{N_{2}O,c}$ (mg-N/g-VSS/h) are the N_2_O accumulation rate and consumption rate measured by the experiment. $R_{N_{2}O,net}$ (mg-N/g-VSS/h) is the net N_2_O accumulation rate, which was attained by subtracting $R_{N_{2}O,c}$ from $R_{N_{2}O,a}$. $R_{N_{2}O,a}$ and $R_{N_{2}O,c}$ were determined using linear regression of the linear part of each concentration profile during the period with the highest linearity. $\Delta C_{N_{2}O,a}$ (mg-N/L) and $\Delta C_{N_{2}O,c}$ (mg-N/L) represent increased and decreased concentrations in N_2_O accumulation and consumption processes, respectively.

**1.3. Calculation of the sulfur-to-nitrogen mass ratio (S/N ratio)**

To evaluate the stoichiometric relationship between sulfide oxidation and nitrate reduction, the sulfur-to-nitrogen (S/N) ratio was calculated based on measured concentrations of sulfate produced and nitrate removed.

When NO_3_^−^ is served as the sole electron acceptor:

$$\frac{S}{N}mass ratio=-\frac{\Delta C_{{{SO}_{4}}^{2-}, t}}{\Delta C_{{{NO}_{3}}^{-},t}+\Delta C_{{{NO}_{2}}^{-},t}}$$

When NO_2_^−^ is served as the sole electron acceptor:

$$\frac{S}{N}mass ratio=-\frac{\Delta C_{{{SO}_{4}}^{2-}, t}}{\Delta C_{{{NO}_{2}}^{-},t}}$$

When NO is served as the sole electron acceptor:

$$\frac{S}{N}mass ratio=-\frac{\Delta C_{{{SO}_{4}}^{2-}, t}}{\Delta C_{{{NO}_{2}}^{-},t}}$$

When N_2_O is served as the sole electron acceptor:

$$\frac{S}{N}mass ratio=-\frac{\Delta C_{{{SO}_{4}}^{2-}, t}}{\Delta C_{N_{2}O,t}}$$

where, $\Delta C_{{NO}_{X},t}$ (mg-N/L) and $\Delta C_{{{SO}_{4}}^{2-}, t}$ (mg-S/L) are the concentration changes in nitrous compounds and sulfate over time.

**1.4. Measurement of the gas concentrations in the headspace and medium**

The gas concentration in the vial headspace was measured by quadruple gas chromatography/mass spectrometry (GC/MS; GCMS-QP2010 Ultra, Shimadzu Corporation, Kyoto, Japan) equipped with a CP-PoraPLOT column (film thickness 10 μm, length 25.0 m, and inner diameter 0.32 mm, Agilent Technologies Japan, Ltd., Tokyo, Japan). The partial pressure of each gas was calculated from the composition ratio and the internal pressure, enabling the calculation of N_2_O concentrations using the gas equation of state (Xu et al., 2025). The concentrations of dissolved N_2_O in the medium were calculated based on the gas-liquid equilibrium using Henry’s constant at 25 ℃. The calculation formula for dissolved gas concentration is as follows:

$$C_{N_{2}O} =(Henry’s constant) \times P_{\mathrm{gas}} \times M_{\mathrm{gas}} \times{10}^{-6}$$

where, $C_{N_{2}O}$ is dissolved N_2_O concentration (mg/L), P_gas_ (kPa) and M_gas_ (mg/mol) are the gaseous partial pressure and molar mass, respectively.

**1.5. 16S rRNA gene amplicon sequencing method**

DNA concentration was measured using the Synergy LX system (Agilent Technologies) in combination with the QuantiFluor dsDNA System (Promega). Library preparation was performed using the 2-step tailed PCR method with the primer sets tabulated in Table S2.

The prepared library concentration was determined using the Synergy H1 system (Agilent Technologies) with the QuantiFluor dsDNA System. The library quality was assessed using the Fragment Analyzer system and the dsDNA 915 Reagent Kit (Agilent Technologies). Sequencing was conducted using the MiSeq system and the MiSeq Reagent Kit v3 (Illumina) under paired-end 2 × 300 bp conditions (Kinh et al., 2017).

**1.6. Metagenomic assembly, binning, and annotation**

The raw reads were quality-filtered and trimmed using fastp v0.23.2 to remove bases with a quality score below Q30, terminal 1 bp from each end, reads shorter than 20 bp, and reads containing ≥10 ambiguous bases (N) or low-quality bases at the 3’ end. Kraken2 v2.0.9 using the Genome Taxonomy Database (GTDB, Release 226) and Bracken v3.1 were first used with the trimmed reads to perform taxonomic profiling of metagenomic reads and obtain an overview of microbial community composition. The processed reads were subsequently used for metagenome-assembled genome (MAG) reconstruction and gene functional analysis, as described below.

In each assembly type, initial binning with MetaBAT2 v2.12.1, MaxBin2 v2.2.6, and CONCOCT v1.0.0, and bin refinement were carried out using the MetaWRAP modules (“binning” and “bin_refinement”). The refined bins from both co- and single-assemblies were further evaluated for genome quality using CheckM2 v0.1.3, and only those with >70% completeness and <5% contamination were retained. The resulting MAGs were dereplicated using dRep v3.4.1 to remove redundancy. Taxonomic annotation of MAGs was performed using GTDB-Tk v2.4.1 (GTDB Release 226, --skip_ani_screen) to assign taxonomy based on the standardized bacterial and archaeal genome taxonomy. Functional annotation of MAGs was carried out using Prodigal v2.6.3 for gene prediction, followed by eggNOG-mapper v2.1.12 and GhostKOALA v3.1 for functional assignments. The functional profiles obtained from GhostKOALA were visualized using KEGG-Decoder v1.2.1. The relative abundance of each MAG was estimated using CoverM v0.4.0 (min-read-aligned-percent 0.75, --min-read-percent-identity 0.95) based on read coverage across bins. Files for data deposition were generated using DFAST v1.2.17 in accordance with repository requirements.

After quality screening and dereplication, we successfully reconstructed 76 MAGs (Completeness >70% and contamination <5%) from S21, S22, S23, and S24 metagenomes. The genome sizes (cumulative length of contigs in MAG bins) and GC contents of the reconstructed MAGs range between 1.60 and 6.05 Mbp and 33.5 and 71.8%, respectively (Table S5). 76 MAGs occupied 64% (S21), 66% (S22), 64% (S23), and 36% (S24) of each total community. Notably, 37 of the 76 MAGs may represent novel species, as their average nucleotide identity (ANI) values were below 95% compared with reference genomes in the Genome Taxonomy Database (GTDB).

For gene functional analysis, open reading frames (ORFs) were predicted from assembled contigs using Prokka v1.14.6, and protein sequences shorter than 30 amino acids were removed prior to clustering and functional annotation. The resulting protein sequences were clustered using CD-HIT v4.8.1 with modified parameters: 95% sequence identity, 90% alignment coverage for the shorter sequence, and local alignment mode to construct a non-redundant gene catalog. Quality-filtered reads were mapped back to the gene catalog using BWA-MEM2 v2.2.1. Filtered alignments were processed with msamtools v1.1.3 to retain high-quality mappings (≥80 bp, ≥95% identity, MAPQ ≥80) and best hits only (https://github.com/arumugamlab/msamtools). Gene abundance was estimated using msamtools with proportional multi-mapping, TPM normalization, and a minimum count threshold of 10. Functional annotation of genes was performed using eggNOG-mapper v2.1.12 (--seed_ortholog_evalue 1e^−5^, --evalue 1e^−5^). Protein sequences were compared against the NCBI non-redundant (nr) protein database using DIAMOND v2.1.9.163 using blastp with an e-value cutoff of 1e−5 and a maximum of one best hit per query.

**1.7. Quantification of gene abundance within metagenome-assembled genomes**

Gene abundance within metagenome-assembled genomes (MAGs) was quantified by mapping quality-controlled metagenomic reads to a non-redundant set of MAG contigs. Paired-end reads were mapped to concatenated MAG reference sequences using BBMap v38.96 (Bushnell, 2014) with a minimum identity threshold of 95% (minid=0.95) and best-hit assignment for multi-mapping reads (ambiguous=best). The resulting SAM files were converted to sorted BAM files using SAMtools v1.15.1 (Li et al., 2009). Gene-level read counts were obtained using featureCounts v2.1.1 (Liao et al., 2014) based on GFF annotations generated by Prokka v1.14.6 (Seemann, 2014), specifying coding sequences (CDS) and locus tags as gene identifiers. Gene abundance was normalized to transcripts per million (TPM) to account for gene length and sequencing depth. TPM values were calculated in R using the dplyr v1.1.4 package (https://dplyr.tidyverse.org). Functional annotation was assigned using GhostKOALA v3.1 (Kanehisa et al., 2016), and genes were classified based on KEGG Orthology (KO) identifiers.

**1.8. Third subculture attempt and exclusion rationale**

A third subculture was also conducted using the same transfer protocol. However, denitrification and sulfur oxidation activities significantly decreased under all four electron-acceptor conditions during this cycle (Figure S1). Under the NO_2_^⁻^-fed condition, the NO_2_^⁻^ concentration remained consistently high (around 60–100 mg-N/L) from day 5 to day 25, with no further decrease, indicating that NO_2_^⁻^ reduction activity had nearly ceased. Sulfide oxidation kinetics also became markedly unstable under the NO- and N_2_O-fed conditions, with non-monotonic patterns inconsistent with the relatively stable performance observed during the first and second subcultures (Supplementary Figure S2). This deterioration primarily resulted from the progressive decline in total biomass across successive transfers: under the strict inorganic, organic carbon-free conditions used in this study, biomass accumulation was inherently limited, and repeated 20% (v/v) transfers gradually reduced viable cell density by the third cycle. Under these biomass-limited conditions, the energetic demands of denitrification under NO_2_⁻, NO, and N_2_O could no longer be sustained, rendering denitrification rates undetectable or highly variable. The third subculture was therefore excluded from all subsequent microbial community, qPCR, and metagenomic analyses, and all results reported in this study correspond exclusively to the first and second subculture stages, during which denitrification and sulfur oxidation activities remained robust and reproducible.

2. **Supplementary Figures and Tables**

**2.1	Supplementary Figures**

**Supplementary Figure 1.** (A) Schematic diagram of the inoculum cultivation experimental setup. (B) DO spot sensor monitoring in batch tests. Nitrogen and sulfur performance during the third subculture period under different electron-acceptor conditions, (C) nitrate, (D) nitrite, (E) nitric oxide, (F) nitrous oxide.

**Supplementary Figure 2.** Nitrogen and sulfur performance during the first and second subculture periods under different electron acceptor conditions involving sulfide. The left column represents the first subculture, while the right column depicts the second subculture. Four electron acceptor conditions correspond to each row: (A) (B) nitrate; (C) (D) nitrite; (E) (F) nitric oxide; (G) (H) nitrous oxide.

**Supplementary Figure 3.** Metagenomic workflow.

**Supplementary Figure 4.** Microbial community compositions and relative abundances with different electron acceptors (NO_3_^−^ (S11, S21); NO_2_^−^ (S12, S22); NO (S13, S23); and N_2_O (S14, S24)) under initial (S0), first subculture (S11-S14), and second subculture (S21-S24) stages: (A) phylum level, (B) class level, and (C) genus level.

**Supplementary Figure 5.** Phylogenetic placement of *Thiobacillus*-affiliated MAGs (MAG10, MAG11, MAG25) within the reconstructed maximum likelihood tree.

**Supplementary Figure 6.** Fold changes in denitrification functional gene copy numbers under different nitrogen and sulfide substrate combinations. (A) *nirS*, (B) *nirK*, (C) clade I *nosZ*, and (D) clade II *nosZ*.

**2.2	Supplementary Tables**

**Supplementary Table 1**. Medium compositions and concentrations applied in this study.

| Component | Media composition | Concentration |
| --- | --- | --- |
| The main chemicals | NaHCO_3_ | 2.0 g/L |
|  | MgCl_2_ | 0.2 g/L |
|  | KH_2_PO_4_ | 2.0 g/L |
| Trace element solution 1.0 mL  (contained per 1 L of distilled water) | ZnCl_2_ | 0.5 g |
|  | NaMoO_4_·2H_2_O | 0.5 g |
|  | MnCl_2_·4H_2_O | 2.5 g |
|  | CuSO_4_ 5H_2_O | 0.1 g |
|  | CoCl_2_·6H_2_O | 0.5 g |
|  | FeCl_2_·4H_2_O | 3.8 g |
|  | HCl | 50.0 mL |

**Supplementary Table 2**. The primer sets for library preparation.

| Step | Primer | Sequence (5’ → 3’) |
| --- | --- | --- |
| 1st PCR | 1st-515f_MIX | ACACTCTTTCCCTACACGACGCTCTTCCGATCT-NNNNN-GTGCCAGCMGCCGCGGTAA |
|  | 1st-806r_MIX | GTGACTGGAGTTCAGACGTGTGCTCTTCCGATCT  NNNNN-GGACTACHVGGGTWTCTAAT |
| 2nd PCR | 2ndF | AATGATACGGCGACCACCGAGATCTACAC-Index2-ACACTCTTTCCCTACACGACGC |
|  | 2ndR | CAAGCAGAAGACGGCATACGAGAT-Index1 GTGACTGGAGTTCAGACGTGTG |

**Supplementary Table 3**. Primer sequences used for qPCR analysis and amplification procedure.

| Primer | Target | Sequence (5′ - 3′) | Amplification protocol | References |
| --- | --- | --- | --- | --- |
| nirS4QF | *nir*S | GTSAACGYSAAGGARACSGG | Initial denaturation at 95 °C for 15 min, followed by 6 cycles of 95 °C for 15 s, 63 °C 30 s, 72 °C 30 s, 80 °C 30 s, and then 40 cycles of 95 °C for 15 s, 58 °C 30 s, 72 °C 30 s, and 80 °C for 30 s. | Throback et al., 2004 |
| nirS4QR |  | GASTTCGGRTGSGTCTTSAYGAA |  |  |
| nirK876-F | *nir*K | ATYGGCGGVCAYGGCGA | Initial denaturation at 95 °C for 15 min, followed by 6 cycles of 95 °C for 15 s, 63 °C 30 s, 72 °C 30 s, 80 °C 30 s, and then 40 cycles of 95 °C for 15 s, 58 °C 30 s, 72 °C 30 s, and 80 °C for 30 s. | Henry and Kenchington, 2004 |
| nirK1040-R |  | GCCTCGATCAGRTTRTGGTT |  |  |
| nosZ2F | clade I *nos*Z | CGCRACGGCAASAAGGTSMSSGT | Initial denaturation at 95 °C for 15 min, followed by 6 cycles of 95 °C for 15 s, 67 °C 30 s, 72 °C 30 s, 80 °C for 30 s, and then 40 cycles of 95 °C for 15 s, 62 °C 30 s, 72 °C 30 s, and 80 °C for 30 s. | Henry et al., 2006 |
| nosZ2R |  | CAKRTGCAKSGCRTGGCAGAA |  |  |
| nosZ-II-F | clade II *nos*Z | CTIGGICCIYTKCAYAC | Initial denaturation at 95 °C for 1 min, followed by 50 cycles of 95 °C for 15 s, 54 °C for 30 s, and 72 °C for 30 s, and 80 °C for 30 s. | Jones et al., 2013 |
| nosZ-II-R |  | GCIGARCARAAITCBGTRC |  |  |

* Reverse complement of 3' Illumina adapter, Golay barcode, Reverse primer pad, Reverse primer linker, Reverse primer (806r)

M = A or C; W = A or T; Y = C or T; S = C or G; R = A or G; K = G or T; H = A or C or T; V = A or C or G; B = C or G or T; I = Inosine; X = Tag sequence

**Supplementary Table 4.** Taxonomy assignment of MAGs using GTDB-Tk.

| MAG ID | Classification |
| --- | --- |
| MAG01 | *d_Bacteria; p_Chloroflexota; c_Anaerolineae; o_Anaerolineales; f_Anaerolineaceae; g_UBA6170; s_* |
| MAG02 | *d_Bacteria; p_Actinomycetota; c_Acidimicrobiia; o_Acidimicrobiales; f_Microtrichaceae; g_SSF14; s_SSF14 sp037045585* |
| MAG03 | *d_Bacteria; p_Bacillota; c_Clostridia; o_Christensenellales; f_CAG-138; g_UBA7707; s_* |
| MAG04 | *d_Bacteria; p_Pseudomonadota; c_Alphaproteobacteria; o_Rhizobiales; f_Rhizobiaceae; g_Shinella; s_* |
| MAG05 | *d_Bacteria; p_Pseudomonadota; c_Gammaproteobacteria; o_Xanthomonadales; f_Xanthomonadaceae; g_Thermomonas; s_Thermomonas sp014678725* |
| MAG06 | *d_Bacteria; p_Actinomycetota; c_Actinomycetes; o_Actinomycetales; f_Dermatophilaceae; g_Phosphoribacter; s_* |
| MAG07 | *d_Bacteria; p_Pseudomonadota; c_Gammaproteobacteria; o_Burkholderiales; f_Rhodocyclaceae; g_Azonexus; s_Azonexus sp040275885* |
| MAG08 | *d_Bacteria; p_Bacillota; c_Desulfotomaculia; o_Desulfotomaculales; f_Desulfocucumaceae; g_BRHc4a; s_* |
| MAG09 | *d_Bacteria; p_Actinomycetota; c_Actinomycetes; o_Actinomycetales; f_Dermatophilaceae; g_Phosphoribacter; s_Phosphoribacter baldrii* |
| MAG10 | *d_Bacteria; p_Pseudomonadota; c_Gammaproteobacteria; o_Burkholderiales; f_Thiobacillaceae; g_Thiobacillus; s_Thiobacillus sp001899775* |
| MAG11 | *d_Bacteria; p_Pseudomonadota; c_Gammaproteobacteria; o_Burkholderiales; f_Thiobacillaceae; g_Thiobacillus; s_* |
| MAG12 | *d_Bacteria; p_Pseudomonadota; c_Alphaproteobacteria; o_Rhodobacterales; f_Rhodobacteraceae; g_Paracoccus; s_Paracoccus versutus* |
| MAG13 | *d_Bacteria; p_Pseudomonadota; c_Gammaproteobacteria; o_Xanthomonadales; f_Rhodanobacteraceae; g_Dokdonella_A; s_Dokdonella_A sp019634845* |
| MAG14 | *d_Bacteria; p_Actinomycetota; c_Acidimicrobiia; o_Acidimicrobiales; f_Microtrichaceae; g_JADJBW01; s_JADJBW01 sp016703525* |
| MAG15 | *d_Bacteria; p_Pseudomonadota; c_Gammaproteobacteria; o_Burkholderiales; f_Aquaspirillaceae; g_Microvirgula; s_Microvirgula aerodenitrificans* |
| MAG16 | *d_Bacteria; p_Bacteroidota; c_Bacteroidia; o_Flavobacteriales; f_Weeksellaceae; g_Moheibacter; s_Moheibacter sp019455045* |
| MAG17 | *d_Bacteria; p_Bacteroidota; c_Bacteroidia; o_Bacteroidales; f_Dysgonomonadaceae; g_JAAYFB01; s_* |
| MAG18 | *d_Bacteria; p_Pseudomonadota; c_Alphaproteobacteria; o_Rhizobiales; f_Rhizobiaceae; g_Brucella; s_Brucella anthropi* |
| MAG19 | *d_Bacteria; p_Pseudomonadota; c_Gammaproteobacteria; o_Enterobacterales; f_Aeromonadaceae; g_Aeromonas; s_Aeromonas hydrophila* |
| MAG20 | *d_Bacteria; p_Pseudomonadota; c_Gammaproteobacteria; o_Xanthomonadales; f_Xanthomonadaceae; g_Pseudoxanthomonas_A; s_* |
| MAG21 | *d_Bacteria; p_Pseudomonadota; c_Gammaproteobacteria; o_Xanthomonadales; f_Xanthomonadaceae; g_Stenotrophomonas; s_Stenotrophomonas acidaminiphila* |
| MAG22 | *d_Bacteria; p_Pseudomonadota; c_Alphaproteobacteria; o_Rhizobiales; f_Rhizobiaceae; g_Shinella; s_Shinella zoogloeoides_A* |
| MAG23 | *d_Bacteria; p_Pseudomonadota; c_Gammaproteobacteria; o_Burkholderiales; f_Burkholderiaceae; g_Alicycliphilus; s_Alicycliphilus denitrificans* |
| MAG24 | *d_Bacteria; p_Pseudomonadota; c_Alphaproteobacteria; o_Rhizobiales; f_Rhizobiaceae; g_Shinella; s_Shinella sp016839145* |
| MAG25 | *d_Bacteria; p_Pseudomonadota; c_Gammaproteobacteria; o_Burkholderiales; f_Thiobacillaceae; g_Thiobacillus; s_Thiobacillus thioparus* |
| MAG26 | *d_Bacteria; p_Bacteroidota; c_Bacteroidia; o_Bacteroidales; f_Dysgonomonadaceae; g_Petrimonas; s_Petrimonas sulfuriphila* |
| MAG27 | *d_Bacteria; p_Pseudomonadota; c_Gammaproteobacteria; o_Burkholderiales; f_Burkholderiaceae; g_Ottowia; s_* |
| MAG28 | *d_Bacteria; p_Pseudomonadota; c_Gammaproteobacteria; o_Xanthomonadales; f_Xanthomonadaceae; g_Denitratimonas; s_Denitratimonas tolerans* |
| MAG29 | *d_Bacteria; p_Pseudomonadota; c_Alphaproteobacteria; o_Micropepsales; f_Micropepsaceae; g_JADZAI01; s_* |
| MAG30 | *d_Bacteria; p_Pseudomonadota; c_Alphaproteobacteria; o_Rhizobiales; f_Ancalomicrobiaceae; g_; s_* |
| MAG31 | *d_Bacteria; p_Chloroflexota; c_Dehalococcoidia; o_Tepidiformales; f_Tepidiformaceae; g_Amarobacillus; s_Amarobacillus elongatus* |
| MAG32 | *d_Bacteria; p_Pseudomonadota; c_Gammaproteobacteria; o_Burkholderiales; f_Rhodocyclaceae; g_Azonexus; s_* |
| MAG33 | *d_Bacteria; p_Planctomycetota; c_Planctomycetia; o_Planctomycetales; f_Planctomycetaceae; g_; s_* |
| MAG34 | *d_Bacteria; p_Chloroflexota; c_Anaerolineae; o_J036; f_Brachytrichaceae; g_Brachythrix; s_Brachythrix sp017998555* |
| MAG35 | *d_Bacteria; p_Pseudomonadota; c_Gammaproteobacteria; o_Burkholderiales; f_Burkholderiaceae; g_Simplicispira; s_Simplicispira sedimenti* |
| MAG36 | *d_Bacteria; p_Pseudomonadota; c_Gammaproteobacteria; o_Pseudomonadales; f_Moraxellaceae; g_Acinetobacter; s_Acinetobacter tandoii* |
| MAG37 | *d_Bacteria; p_Actinomycetota; c_Acidimicrobiia; o_Acidimicrobiales; f_Microtrichaceae; g_Microthrix; s_Microthrix parvicella* |
| MAG38 | *d_Bacteria; p_Pseudomonadota; c_Gammaproteobacteria; o_Burkholderiales; f_Rhodocyclaceae; g_Sulfuricystis; s_* |
| MAG39 | *d_Bacteria; p_Pseudomonadota; c_Gammaproteobacteria; o_Burkholderiales; f_Casimicrobiaceae; g_CAIWHR01; s_* |
| MAG40 | *d_Bacteria; p_Pseudomonadota; c_Gammaproteobacteria; o_Chromatiales; f_Chromatiaceae; g_; s_* |
| MAG41 | *d_Bacteria; p_Chloroflexota; c_Chloroflexia; o_Chloroflexales; f_Roseiflexaceae; g_Kouleothrix; s_Kouleothrix ribensis* |
| MAG42 | *d_Bacteria; p_Pseudomonadota; c_Alphaproteobacteria; o_Sphingomonadales; f_Sphingomonadaceae; g_Novosphingobium; s_* |
| MAG43 | *d_Bacteria; p_Acidobacteriota; c_Holophagae; o_Holophagales; f_Holophagaceae; g_JACQZU01; s_* |
| MAG44 | *d_Bacteria; p_Bacillota; c_Clostridia; o_Peptostreptococcales; f_Acidaminobacteraceae; g_Fusibacter_C; s_* |
| MAG45 | *d_Bacteria; p_Pseudomonadota; c_Gammaproteobacteria; o_Burkholderiales; f_Burkholderiaceae; g_Zeimonas; s_Zeimonas sp003576795* |
| MAG46 | *d_Bacteria; p_Bacteroidota_A; c_Chlorobiia; o_Chlorobiales; f_Chlorobiaceae; g_Chlorobium; s_Chlorobium sp013334435* |
| MAG47 | *d_Bacteria; p_Planctomycetota; c_Planctomycetia; o_Planctomycetales; f_Planctomycetaceae; g_TH-plancto1; s_* |
| MAG48 | *d_Bacteria; p_Cyanobacteriota; c_Vampirovibrionia; o_Obscuribacterales; f_Obscuribacteraceae; g_Obscuribacter; s_* |
| MAG49 | *d_Bacteria; p_Verrucomicrobiota; c_Verrucomicrobiia; o_Limisphaerales; f_UBA11358; g_JADLHS01; s_JADLHS01 sp020848635* |
| MAG50 | *d_Bacteria; p_Actinomycetota; c_Acidimicrobiia; o_Acidimicrobiales; f_Ilumatobacteraceae; g_UBA668; s_UBA668 sp002299395* |
| MAG51 | *d_Bacteria; p_Bacteroidota_A; c_Ignavibacteria; o_SJA-28; f_OLB5; g_OLB5; s_* |
| MAG52 | *d_Bacteria; p_Chloroflexota; c_Anaerolineae; o_Caldilineales; f_Caldilineaceae; g_Caldilinea; s_Caldilinea saccharophila* |
| MAG53 | *d_Bacteria; p_Actinomycetota; c_Actinomycetes; o_Actinomycetales; f_Dermatophilaceae; g_Lutibacillus; s_* |
| MAG54 | *d_Bacteria; p_Acidobacteriota; c_Blastocatellia; o_Pyrinomonadales; f_Pyrinomonadaceae; g_OLB17; s_* |
| MAG55 | *d_Bacteria; p_Pseudomonadota; c_Gammaproteobacteria; o_Burkholderiales; f_Burkholderiaceae; g_Comamonas; s_Comamonas testosteroni_H* |
| MAG56 | *d_Bacteria; p_Pseudomonadota; c_Gammaproteobacteria; o_Burkholderiales; f_Rhodocyclaceae; g_Accumulibacter; s_Accumulibacter sp016789495* |
| MAG57 | *d_Bacteria; p_Pseudomonadota; c_Gammaproteobacteria; o_Burkholderiales; f_Burkholderiaceae; g_Rubrivivax; s_Rubrivivax sp963205115* |
| MAG58 | *d_Bacteria; p_Actinomycetota; c_Thermoleophilia; o_Miltoncostaeales; f_Miltoncostaeaceae; g_CALMEC01; s_* |
| MAG59 | *d_Bacteria; p_Acidobacteriota; c_Thermoanaerobaculia; o_UBA5066; f_UBA5066; g_UBA5066; s_* |
| MAG60 | *d_Bacteria; p_Pseudomonadota; c_Gammaproteobacteria; o_Burkholderiales; f_Burkholderiaceae; g_Zeimonas; s_Zeimonas sp937872345* |
| MAG61 | *d_Bacteria; p_Actinomycetota; c_Acidimicrobiia; o_Acidimicrobiales; f_Microtrichaceae; g_UBA11034; s_* |
| MAG62 | *d_Bacteria; p_Pseudomonadota; c_Gammaproteobacteria; o_Burkholderiales; f_Burkholderiaceae; g_Aquabacterium_A; s_* |
| MAG63 | *d_Bacteria; p_Actinomycetota; c_Acidimicrobiia; o_Acidimicrobiales; f_Iamiaceae; g_; s_* |
| MAG64 | *d_Bacteria; p_Actinomycetota; c_Actinomycetes; o_Propionibacteriales; f_Nocardioidaceae; g_Marmoricola; s_Marmoricola sp937868455* |
| MAG65 | *d_Bacteria; p_Pseudomonadota; c_Alphaproteobacteria; o_Rhizobiales; f_Ancalomicrobiaceae; g_Pinisolibacter; s_* |
| MAG66 | *d_Bacteria; p_Pseudomonadota; c_Gammaproteobacteria; o_Burkholderiales; f_Rhodocyclaceae; g_Thauera; s_Thauera terpenica* |
| MAG67 | *d_Bacteria; p_Bacillota; c_Clostridia; o_Tissierellales; f_Sedimentibacteraceae; g_Sedimentibacter; s_Sedimentibacter sp023425285* |
| MAG68 | *d_Bacteria; p_Pseudomonadota; c_Alphaproteobacteria; o_Rhizobiales; f_Hyphomicrobiaceae; g_Hyphomicrobium_B; s_Hyphomicrobium_B sp016716675* |
| MAG69 | *d_Bacteria; p_Actinomycetota; c_Acidimicrobiia; o_Acidimicrobiales; f_Microtrichaceae; g_; s_* |
| MAG70 | *d_Bacteria; p_Actinomycetota; c_Acidimicrobiia; o_Acidimicrobiales; f_Ilumatobacteraceae; g_VFMC01; s_* |
| MAG71 | *d_Bacteria; p_Pseudomonadota; c_Alphaproteobacteria; o_Micropepsales; f_Micropepsaceae; g_JADZAI01; s_* |
| MAG72 | *d_Bacteria; p_Planctomycetota; c_Planctomycetia; o_Pirellulales; f_Pirellulaceae; g_JBEISR01; s_* |
| MAG73 | *d_Bacteria; p_Pseudomonadota; c_Alphaproteobacteria; o_Rhizobiales; f_Beijerinckiaceae; g_; s_* |
| MAG74 | *d_Bacteria; p_Bacteroidota; c_Bacteroidia; o_Flavobacteriales; f_Weeksellaceae; g_Cloacibacterium; s_Cloacibacterium normanense* |
| MAG75 | *d_Bacteria; p_Planctomycetota; c_Planctomycetia; o_Gemmatales; f_Gemmataceae; g_DASXPC01; s_* |
| MAG76 | *d_Bacteria; p_Actinomycetota; c_Acidimicrobiia; o_Acidimicrobiales; f_WLNW01; g_WLNW01; s_* |

**Supplementary Table 5.** Properties of the MAGs reconstructed from the metagenomes**.**

| MAG ID | Total Sequence Length (bp) | Number of Sequences | GC content (%) | Genome QC (CheckM2) | | Relative abundance (CoverM) | | | |
| --- | --- | --- | --- | --- | --- | --- | --- | --- | --- |
|  |  |  |  | Completeness (%) | Contamination (%) | NO_3_^-^ | NO_2_^-^ | NO | N_2_O |
| MAG01 | 2624678 | 58 | 40.8 | 85.64 | 0.5 | 0.45 | 0.03 | 0.00 | 0.00 |
| MAG02 | 3502887 | 613 | 68.9 | 78.39 | 0.64 | 0.17 | 0.21 | 0.18 | 1.56 |
| MAG03 | 3068321 | 37 | 57.4 | 95.21 | 1.04 | 0.49 | 0.04 | 0.04 | 0.02 |
| MAG04 | 3966739 | 308 | 65.8 | 83.09 | 1.98 | 0.54 | 0.00 | 0.01 | 0.03 |
| MAG05 | 2341747 | 431 | 69.3 | 82.59 | 1.74 | 1.00 | 0.03 | 0.03 | 0.03 |
| MAG06 | 3355201 | 146 | 69.4 | 92.61 | 0.59 | 0.33 | 0.22 | 0.23 | 2.07 |
| MAG07 | 2604681 | 276 | 61.9 | 84.17 | 4.04 | 0.21 | 0.02 | 0.05 | 1.22 |
| MAG08 | 4082137 | 420 | 53.8 | 99.9 | 3.06 | 0.29 | 0.02 | 0.00 | 0.00 |
| MAG09 | 3525237 | 340 | 69.3 | 97.13 | 4.32 | 0.19 | 0.15 | 0.15 | 1.50 |
| MAG10 | 2366267 | 416 | 65.7 | 80.3 | 0.56 | 51.11 | 56.44 | 58.66 | 1.00 |
| MAG11 | 2465969 | 9 | 66.9 | 84.03 | 0.2 | 1.16 | 0.06 | 0.07 | 0.02 |
| MAG12 | 4206445 | 78 | 68.5 | 99.6 | 0 | 1.59 | 0.00 | 0.00 | 0.00 |
| MAG13 | 3165114 | 54 | 68.9 | 87.85 | 0.14 | 0.01 | 0.20 | 0.00 | 0.01 |
| MAG14 | 3269642 | 380 | 69.8 | 85.33 | 0.83 | 0.08 | 0.11 | 0.09 | 0.71 |
| MAG15 | 3745030 | 159 | 64.7 | 99.69 | 2.63 | 0.00 | 0.14 | 0.00 | 0.06 |
| MAG16 | 2854719 | 77 | 40.1 | 99.87 | 0.13 | 0.00 | 0.66 | 0.01 | 0.10 |
| MAG17 | 3768959 | 48 | 45.2 | 99.24 | 1.19 | 0.00 | 0.29 | 0.01 | 0.00 |
| MAG18 | 4570436 | 32 | 56.1 | 98.53 | 0.29 | 0.02 | 0.16 | 0.02 | 0.04 |
| MAG19 | 4714752 | 25 | 61.6 | 100 | 0.34 | 0.05 | 0.20 | 0.04 | 0.87 |
| MAG20 | 3824428 | 186 | 67.9 | 91.33 | 1.66 | 0.02 | 0.14 | 0.00 | 0.00 |
| MAG21 | 3847673 | 35 | 69.2 | 100 | 0.18 | 2.34 | 1.26 | 0.04 | 0.30 |
| MAG22 | 4781443 | 128 | 65.8 | 99.65 | 3.09 | 0.02 | 0.14 | 0.02 | 0.03 |
| MAG23 | 4596508 | 39 | 69 | 99.99 | 0.2 | 1.20 | 0.06 | 0.70 | 0.09 |
| MAG24 | 4131199 | 369 | 66.1 | 80.97 | 4.2 | 0.12 | 0.01 | 0.11 | 0.18 |
| MAG25 | 2639174 | 10 | 62.4 | 77.2 | 0.75 | 0.04 | 0.11 | 0.57 | 1.58 |
| MAG26 | 3356145 | 203 | 44.5 | 99.9 | 0.29 | 0.70 | 0.28 | 0.11 | 0.01 |
| MAG27 | 3399336 | 335 | 67 | 95.96 | 4.32 | 0.31 | 0.14 | 0.21 | 2.19 |
| MAG28 | 3016663 | 28 | 68.6 | 99.99 | 0.07 | 0.01 | 0.00 | 1.56 | 0.02 |
| MAG29 | 3278086 | 237 | 69.3 | 94.05 | 2.93 | 0.14 | 0.08 | 0.10 | 0.68 |
| MAG30 | 3662696 | 54 | 65.8 | 98.74 | 0.37 | 0.02 | 0.01 | 0.05 | 0.41 |
| MAG31 | 2998903 | 549 | 69.1 | 87.22 | 3.6 | 0.02 | 0.01 | 0.01 | 0.20 |
| MAG32 | 3701698 | 78 | 64.9 | 87.17 | 3.99 | 0.01 | 0.00 | 0.01 | 1.39 |
| MAG33 | 5245916 | 494 | 57.7 | 99.25 | 2.63 | 0.02 | 0.04 | 0.03 | 0.20 |
| MAG34 | 4393784 | 761 | 65.1 | 88.06 | 2.92 | 0.03 | 0.01 | 0.01 | 0.45 |
| MAG35 | 3619439 | 59 | 65.6 | 100 | 0.08 | 0.04 | 0.01 | 0.01 | 1.87 |
| MAG36 | 3000967 | 617 | 40.8 | 85.49 | 4.33 | 0.00 | 0.00 | 0.00 | 0.14 |
| MAG37 | 3631393 | 331 | 66.8 | 93.99 | 0.64 | 0.11 | 0.17 | 0.14 | 1.06 |
| MAG38 | 2943319 | 55 | 63 | 100 | 0.42 | 0.00 | 0.00 | 0.02 | 4.48 |
| MAG39 | 4860899 | 167 | 69.8 | 97.73 | 1.3 | 0.02 | 0.02 | 0.02 | 0.37 |
| MAG40 | 3695212 | 321 | 63.2 | 100 | 1.53 | 0.02 | 0.01 | 0.01 | 0.21 |
| MAG41 | 4298634 | 1313 | 65.8 | 81.23 | 3.53 | 0.01 | 0.00 | 0.01 | 0.14 |
| MAG42 | 3165002 | 74 | 66.9 | 99.94 | 0.83 | 0.08 | 0.07 | 0.09 | 0.38 |
| MAG43 | 3364708 | 572 | 70.5 | 83 | 1.46 | 0.03 | 0.02 | 0.01 | 0.82 |
| MAG44 | 3510729 | 243 | 35 | 95.3 | 1.97 | 0.00 | 0.00 | 0.00 | 0.30 |
| MAG45 | 3464516 | 233 | 69.3 | 78.55 | 1.97 | 0.06 | 0.12 | 0.00 | 0.00 |
| MAG46 | 2555453 | 98 | 54.8 | 99.99 | 0.16 | 0.04 | 0.03 | 0.02 | 0.24 |
| MAG47 | 5148289 | 548 | 59 | 83.39 | 2.51 | 0.01 | 0.01 | 0.01 | 0.10 |
| MAG48 | 5003673 | 1474 | 48.1 | 74.21 | 1.66 | 0.01 | 0.00 | 0.00 | 0.10 |
| MAG49 | 4724097 | 715 | 60.1 | 82.6 | 2.59 | 0.02 | 0.00 | 0.00 | 0.12 |
| MAG50 | 4053031 | 130 | 68.4 | 97.58 | 0.39 | 0.07 | 0.07 | 0.08 | 0.60 |
| MAG51 | 3030148 | 543 | 38.6 | 84.21 | 3.01 | 0.01 | 0.01 | 0.01 | 0.08 |
| MAG52 | 4658070 | 552 | 60.8 | 87.68 | 4.11 | 0.03 | 0.01 | 0.01 | 0.16 |
| MAG53 | 2609285 | 463 | 70.4 | 76.73 | 2.11 | 0.03 | 0.03 | 0.07 | 0.28 |
| MAG54 | 4824764 | 330 | 53.5 | 90.86 | 1.94 | 0.01 | 0.02 | 0.01 | 0.10 |
| MAG55 | 4142354 | 492 | 62.4 | 83.79 | 0.55 | 0.00 | 0.00 | 0.00 | 0.35 |
| MAG56 | 3268667 | 184 | 60.2 | 100 | 4.47 | 0.05 | 0.04 | 0.04 | 0.38 |
| MAG57 | 5236086 | 243 | 68.8 | 88.25 | 3.93 | 0.03 | 0.02 | 0.04 | 0.21 |
| MAG58 | 2330968 | 288 | 65.9 | 85.39 | 2.49 | 0.03 | 0.04 | 0.03 | 0.31 |
| MAG59 | 4647162 | 258 | 71.8 | 96.95 | 1.9 | 0.20 | 0.00 | 0.00 | 0.04 |
| MAG60 | 4077431 | 50 | 70.1 | 91.03 | 2.67 | 0.05 | 3.68 | 0.02 | 0.04 |
| MAG61 | 3837838 | 199 | 59.8 | 95.63 | 0.93 | 0.01 | 0.01 | 0.01 | 0.13 |
| MAG62 | 4510776 | 362 | 69 | 88.2 | 3.79 | 0.03 | 0.01 | 0.01 | 0.22 |
| MAG63 | 3102426 | 886 | 70.5 | 76.87 | 1.81 | 0.01 | 0.01 | 0.02 | 0.09 |
| MAG64 | 2520326 | 379 | 61.9 | 92.67 | 3.26 | 0.02 | 0.01 | 0.01 | 0.15 |
| MAG65 | 3664177 | 540 | 70.2 | 89.55 | 4.21 | 0.00 | 0.00 | 0.00 | 0.72 |
| MAG66 | 2272367 | 298 | 65 | 70.93 | 1.75 | 0.02 | 0.07 | 0.00 | 0.04 |
| MAG67 | 3250737 | 114 | 37.8 | 99.5 | 1.9 | 0.01 | 0.11 | 0.00 | 0.00 |
| MAG68 | 3269612 | 185 | 60.5 | 99.18 | 3.38 | 0.06 | 0.04 | 0.03 | 0.18 |
| MAG69 | 3061493 | 443 | 59.1 | 92.5 | 1.5 | 0.05 | 0.06 | 0.05 | 1.07 |
| MAG70 | 4139162 | 249 | 70.8 | 79.33 | 1.37 | 0.02 | 0.03 | 0.03 | 0.24 |
| MAG71 | 3311273 | 23 | 66.1 | 100 | 0.24 | 0.38 | 0.25 | 0.28 | 2.12 |
| MAG72 | 6049814 | 932 | 51.4 | 82.75 | 2.17 | 0.01 | 0.01 | 0.01 | 0.09 |
| MAG73 | 3210793 | 114 | 67 | 100 | 0.17 | 0.03 | 0.03 | 0.04 | 0.28 |
| MAG74 | 1602726 | 880 | 33.5 | 71.19 | 3.22 | 0.01 | 0.00 | 0.00 | 0.09 |
| MAG75 | 5557942 | 548 | 55.5 | 92.98 | 1.64 | 0.01 | 0.01 | 0.01 | 0.11 |
| MAG76 | 3809478 | 287 | 70.6 | 72.89 | 0.57 | 0.01 | 0.01 | 0.01 | 0.17 |

**Supplementary Table 6** KEGG metabolic pathway completeness scores for three *Thiobacillus* MAGs.

| KEGG metabolic pathway | MAG10 | MAG11 | MAG25 |
| --- | --- | --- | --- |
| glycolysis | 0.44 | 0.67 | 0.78 |
| gluconeogenesis | 0.56 | 0.78 | 0.89 |
| TCA Cycle | 0.38 | 0.50 | 0.38 |
| NADH-quinone oxidoreductase | 0.70 | 0.98 | 0.98 |
| F-type ATPase | 0.88 | 1.00 | 1.00 |
| V-type ATPase | 0.11 | 0.00 | 0.22 |
| Cytochrome c oxidase | 0.00 | 0.75 | 0.00 |
| Ubiquinol-cytochrome c reductase | 0.99 | 0.00 | 0.00 |
| Cytochrome c oxidase, cbb3-type | 0.25 | 0.75 | 0.25 |
| Cytochrome bd complex | 0.00 | 1.00 | 1.00 |
| RuBisCo | 1.00 | 1.00 | 1.00 |
| CBB Cycle | 0.25 | 0.91 | 0.92 |
| 3-Hydroxypropionate Bicycle | 0.12 | 0.12 | 0.06 |
| 4-Hydroxybutyrate/3-hydroxypropionate | 0.10 | 0.00 | 0.00 |
| glucoamylase | 0.00 | 1.00 | 0.00 |
| D-galacturonate epimerase | 1.00 | 1.00 | 1.00 |
| beta-N-acetylhexosaminidase | 1.00 | 1.00 | 0.00 |
| nitrite oxidation | 1.00 | 0.00 | 1.00 |
| dissimilatory nitrate reduction | 1.00 | 1.00 | 1.00 |
| nitrite reduction | 1.00 | 1.00 | 1.00 |
| nitric oxide reduction | 1.00 | 1.00 | 0.00 |
| nitrous-oxide reduction | 1.00 | 0.00 | 1.00 |
| dissimilatory sulfate < > APS | 1.00 | 1.00 | 1.00 |
| dissimilatory sulfite < > APS | 1.00 | 1.00 | 1.00 |
| dissimilatory sulfite < > sulfide | 1.00 | 1.00 | 1.00 |
| thiosulfate oxidation | 0.64 | 0.80 | 0.16 |
| alt thiosulfate oxidation tsdA | 0.00 | 0.00 | 1.00 |
| sulfite dehydrogenase (quinone) | 0.00 | 0.66 | 0.00 |
| sulfide oxidation | 1.00 | 1.00 | 1.00 |
| sulfur assimilation | 0.00 | 0.00 | 1.00 |
| NiFe hydrogenase Hyd-1 | 0.66 | 0.00 | 0.66 |
| thiamin biosynthesis | 0.73 | 0.64 | 0.73 |
| riboflavin biosynthesis | 0.00 | 1.00 | 1.00 |
| cobalamin biosynthesis | 0.25 | 0.38 | 0.38 |
| transporter: urea | 0.20 | 0.00 | 0.00 |
| transporter: phosphate | 1.00 | 1.00 | 1.00 |
| Flagellum | 0.78 | 0.87 | 1.00 |
| Chemotaxis | 0.88 | 0.88 | 0.75 |
| Methanogenesis via CO2 | 0.00 | 0.20 | 0.25 |
| Coenzyme B/Coenzyme M regeneration | 0.20 | 0.20 | 0.20 |
| Retinal biosynthesis | 0.25 | 0.25 | 0.25 |
| Retinal from apo-carotenals | 0.00 | 0.00 | 0.00 |
| Entner-Doudoroff Pathway | 0.25 | 0.50 | 0.50 |
| Mixed acid: Formate to CO2 & H2 | 0.00 | 0.50 | 0.67 |
| Mixed acid: Acetate | 0.50 | 0.50 | 0.50 |
| Biofilm PGA Synthesis protein | 0.25 | 0.00 | 0.00 |
| Competence-related core components | 0.00 | 0.14 | 0.14 |
| Anaplerotic genes | 0.50 | 0.50 | 0.50 |
| Type I Secretion | 0.33 | 0.99 | 0.99 |
| Type III Secretion | 0.00 | 0.00 | 0.00 |
| Type II Secretion | 0.54 | 0.54 | 0.31 |
| Type VI Secretion | 0.00 | 0.11 | 0.00 |
| Sec-SRP | 0.00 | 0.83 | 0.83 |
| Twin Arginine Targeting | 0.50 | 0.75 | 0.75 |
| Serine pathway/formaldehyde assimilation | 0.50 | 0.50 | 0.40 |
| Arsenic reduction | 0.75 | 0.75 | 0.75 |
| Cobalt transporter CbiMQ | 0.00 | 0.00 | 0.00 |
| Cobalt transporter CbtA | 0.00 | 0.00 | 0.00 |
| Cobalt transporter CorA | 0.00 | 1.00 | 1.00 |
| Nickel ABC-type substrate-binding NikA | 0.00 | 0.00 | 0.00 |
| Copper transporter CopA | 1.00 | 1.00 | 1.00 |
| Ferrous iron transporter FeoB | 0.00 | 1.00 | 1.00 |
| Ferric iron ABC-type substrate-binding AfuA | 0.00 | 1.00 | 1.00 |
| Fe-Mn transporter MntH | 1.00 | 0.00 | 0.00 |
| histidine | 1.00 | 1.00 | 1.00 |
| arginine | 1.00 | 1.00 | 0.00 |
| lysine | 1.00 | 1.00 | 0.00 |
| serine | 1.00 | 1.00 | 1.00 |
| threonine | 1.00 | 1.00 | 0.00 |
| asparagine | 1.00 | 1.00 | 1.00 |
| glutamine | 1.00 | 1.00 | 1.00 |
| cysteine | 1.00 | 1.00 | 1.00 |
| glycine | 1.00 | 1.00 | 1.00 |
| proline | 1.00 | 1.00 | 1.00 |
| alanine | 1.00 | 1.00 | 0.00 |
| valine | 0.83 | 0.83 | 0.83 |
| methionine | 1.00 | 1.00 | 1.00 |
| phenylalanine | 0.00 | 0.00 | 0.00 |
| isoleucine | 0.83 | 0.83 | 0.83 |
| leucine | 0.75 | 0.50 | 1.00 |
| tryptophan | 0.50 | 0.00 | 1.00 |
| tyrosine | 0.00 | 0.00 | 0.00 |
| aspartate | 0.00 | 1.00 | 1.00 |
| glutamate | 0.00 | 1.00 | 1.00 |
| PET degradation | 0.00 | 0.00 | 0.00 |
| starch/glycogen synthesis | 0.99 | 0.99 | 0.99 |
| starch/glycogen degradation | 1.00 | 1.00 | 1.00 |
| polyhydroxybutyrate synthesis | 0.00 | 0.00 | 0.00 |
| bidirectional polyphosphate | 0.50 | 1.00 | 1.00 |
| carotenoids backbone biosynthesis | 0.40 | 0.40 | 0.40 |
| end-product astaxanthin | 0.00 | 0.00 | 0.00 |
| end-product nostoxanthin | 0.00 | 0.00 | 0.00 |
| end-product zeaxanthin diglucoside | 0.00 | 0.00 | 0.00 |
| end-product myxoxanthophylls | 0.00 | 0.00 | 0.00 |
| staphyloaxanthin biosynthesis | 0.00 | 0.00 | 0.00 |
| mevalonate pathway | 0.00 | 0.00 | 0.00 |
| MEP-DOXP pathway | 0.71 | 0.85 | 0.57 |

**References**

Bushnell, B. (2014). BBMap: A Fast, Accurate, Splice-Aware Aligner. Available at: https://escholarship.org/uc/item/1h3515gn (Accessed April 14, 2026).

Kanehisa, M., Sato, Y., and Morishima, K. (2016). BlastKOALA and GhostKOALA: KEGG Tools for Functional Characterization of Genome and Metagenome Sequences. *Journal of Molecular Biology* 428, 726–731. doi: 10.1016/j.jmb.2015.11.006

Koenig, A. (2001). Kinetic model of autotrophic denitrification in sulphur packed-bed reactors. *Water Research* 35, 1969–1978. doi: 10.1016/S0043-1354(00)00483-8

Li, H., Handsaker, B., Wysoker, A., Fennell, T., Ruan, J., Homer, N., et al. (2009). The Sequence Alignment/Map format and SAMtools. *Bioinformatics* 25, 2078–2079. doi: 10.1093/bioinformatics/btp352

Liao, Y., Smyth, G. K., and Shi, W. (2014). featureCounts: an efficient general purpose program for assigning sequence reads to genomic features. *Bioinformatics* 30, 923–930. doi: 10.1093/bioinformatics/btt656

Lin, S., Mackey, H. R., Hao, T., Guo, G., van Loosdrecht, M. C. M., and Chen, G. (2018). Biological sulfur oxidation in wastewater treatment: A review of emerging opportunities. *Water Research* 143, 399–415. doi: 10.1016/j.watres.2018.06.051

Oberoi, A. S., Huang, H., Khanal, S. K., Sun, L., and Lu, H. (2021). Electron distribution in sulfur-driven autotrophic denitrification under different electron donor and acceptor feeding schemes. *Chem. Eng. J.* 404, 126486. doi: 10.1016/j.cej.2020.126486

Seemann, T. (2014). Prokka: rapid prokaryotic genome annotation. *Bioinformatics* 30, 2068–2069. doi: 10.1093/bioinformatics/btu153

Xu, T., Yasuda, S., Oba, K., Kuroiwa, M., Riya, S., Zhou, Y., et al. (2025). Single cell protein production potential of enriched microbial populations from rice paddy soils and roots: Insights into protein yield enhancement by Methylophilaceae. *Bioresource Technology* 427, 132397. doi: 10.1016/j.biortech.2025.132397

Xu, X., Chen, C., Wang, A., Guo, H., Yuan, Y., Lee, D.-J., et al. (2014). Kinetics of nitrate and sulfate removal using a mixed microbial culture with or without limited-oxygen fed. *Appl Microbiol Biotechnol* 98, 6115–6124. doi: 10.1007/s00253-014-5642-6
